# Structural and Functional Insight into the Mechanism of *Bacillus subtilis* 6S-1 RNA Release from RNA Polymerase

**DOI:** 10.3390/ncrna8010020

**Published:** 2022-02-16

**Authors:** Sweetha Ganapathy, Philipp G. Hoch, Marcus Lechner, Malte Bussiek, Roland K. Hartmann

**Affiliations:** 1 Institut für Pharmazeutische Chemie, Philipps-Universität Marburg, 35037 Marburg, Germany; sweetha.ganapathy@pharmazie.uni-marburg.de (S.G.); phihoch@gmx.net (P.G.H.); 2 Zentrum für Synthetische Mikrobiologie, Philipps-Universität Marburg, 35032 Marburg, Germany; lechner@staff.uni-marburg.de; 3 Institute of Biology, University of Kassel, 34132 Kassel, Germany; m.bussiek@web.de

**Keywords:** *Bacillus subtilis* 6S-1 RNA, truncated and mutated derivatives, 6S-1 RNA:σ^A^-RNAP release kinetics, product RNA (pRNA) transcription, gel shift assays, locked nucleic acid derivatives (pLNAs) of pRNA oligomers, atomic force microscopy

## Abstract

Here we investigated the refolding of *Bacillus subtilis* 6S-1 RNA and its release from σ^A^-RNA polymerase (σ^A^-RNAP) in vitro using truncated and mutated 6S-1 RNA variants. Truncated 6S-1 RNAs, only consisting of the central bubble (CB) flanked by two short helical arms, can still traverse the mechanistic 6S RNA cycle in vitro despite ~10-fold reduced σ^A^-RNAP affinity. This indicates that the RNA’s extended helical arms including the ‘−35′-like region are not required for basic 6S-1 RNA functionality. The role of the ‘central bubble collapse helix’ (CBCH) in pRNA-induced refolding and release of 6S-1 RNA from σ^A^-RNAP was studied by stabilizing mutations. This also revealed base identities in the 5’-part of the CB (5’-CB), upstream of the pRNA transcription start site (nt 40), that impact ground state binding of 6S-1 RNA to σ^A^-RNAP. Stabilization of the CBCH by the C44/45 double mutation shifted the pRNA length pattern to shorter pRNAs and, combined with a weakened P2 helix, resulted in more effective release from RNAP. We conclude that formation of the CBCH supports pRNA-induced 6S-1 RNA refolding and release. Our mutational analysis also unveiled that formation of a second short hairpin in the 3′-CB is detrimental to 6S-1 RNA release. Furthermore, an LNA mimic of a pRNA as short as 6 nt, when annealed to 6S-1 RNA, retarded the RNA’s gel mobility and interfered with σ^A^-RNAP binding. This effect incrementally increased with pLNA 7- and 8-mers, suggesting that restricted conformational flexibility introduced into the 5’-CB by base pairing with pRNAs prevents 6S-1 RNA from adopting an elongated shape. Accordingly, atomic force microscopy of free 6S-1 RNA versus 6S-1:pLNA 8- and 14-mer complexes revealed that 6S-1:pRNA hybrid structures, on average, adopt a more compact structure than 6S-1 RNA alone. Overall, our findings also illustrate that the wild-type 6S-1 RNA sequence and structure ensures an optimal balance of the different functional aspects involved in the mechanistic cycle of 6S-1 RNA.

## 1. Introduction

**Figure 1:**
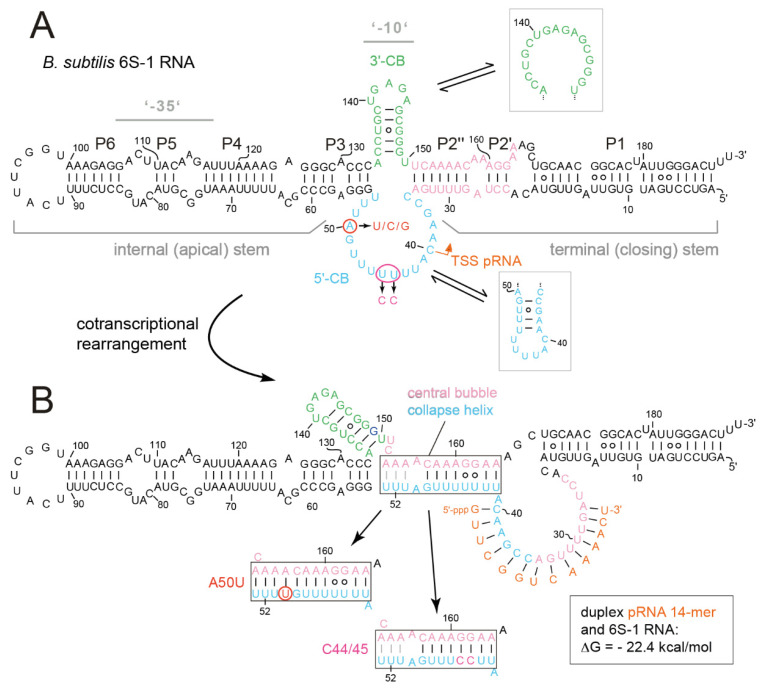
(**A**) Secondary structure of *B. subtilis* 6S-1 RNA in its free state (top) and (**B**) after the structural rearrangement (bottom) induced by transcription of a pRNA 14-mer (orange) that remains stably bound to 6S-1 RNA. The structures were inferred from in-solution probing data [12]. (**A**) The 5’- and 3’-CB (Central Bubble) regions are highlighted in light blue and green, respectively; the orange arrow indicates the transcription start site (TSS) for pRNA synthesis by σ^A^-RNAP. Helix P2 (composed of elements P2’ and P2’’), marked in pink, is disrupted upon pRNA synthesis. Probing data [12] suggested that a hairpin structure forms in the 3’-CB in the free state (in line with RNAfold prediction), where it is in equilibrium with the open conformation; probing suggested that this hairpin is stabilized in the pRNA-bound, rearranged structure ([12]; panel B). RNAfold also predicts weak (transient) base-pairing within the 5’-CB (framed light blue hairpin structure), although this was not confirmed by structure probing [12]. Consensus secondary structure analysis of 14 Firmicutes 6S-1 type RNAs by mlocarna and RNAalifold predicted the 3’-CB hairpin and the open conformation of the 5’-CB [13]. Gray horizontal lines mark the structural elements corresponding to −35 and −10 elements of open DNA promoters, tentatively assigned in analogy to the *E. coli* system. (**B**) Structural probing was also consistent with formation of the so-called ‘central bubble collapse helix’ (CBCH, boxed) that forms between nucleotides of the 5’-CB and 3’-strand of the P2 region [12]. RNAfold dot plot analysis predicts that the three A:U bp (U51-53 and A153-155) are the weakest part of the CBCH and may, thus, form only transiently (highlighted by gray lines connecting the pairing bases), particularly in the wt and C44/45 structures.

Bacterial 6S RNAs are non-coding RNAs (ncRNA) of ~200 nt that form a rod-shaped secondary structure with a relatively unstructured region in the center (termed central bubble) that is flanked by two, non-continuously helical arms: the terminal (closing) stem formed by the RNA’s 5′- and 3′-proximal sequences and the internal (apical) stem capped by a loop (Figure 1 and Figure S1). The helical arms are not perfectly base-paired, but they are interspersed with short internal loops and bulges. The sequence identity of 6S RNAs is limited and only the inclusion of secondary structure conservation (covariance models) allowed their identification as members of the same ncRNA family [1]. The sequence identity between *Bacillus subtilis* 6S-1 RNA and *Escherichia coli* 6S RNA is 38 to 45%, depending on the type of alignment (Figure S1B). The 6S RNAs bind to the active site of RNA polymerase (RNAP) holoenzymes in complex with the major (housekeeping) sigma factor, σ^70^ and σ^A^ in the model organisms *E. coli* and *B. subtilis*, respectively. This results in differential and complex transcription inhibition effects at σ^70^/σ^A^-dependent promoters [2,3,4,5,6,7] and, as a consequence, in global modulation of transcription. A single 6S RNA gene is found in the vast majority of bacterial genomes sequenced so far; some bacteria such as *B. subtilis* even express two 6S paralogs (6S-1 and 6S-2 RNA). A bioinformatic analysis predicted more than 1700 6S RNAs in >1600 bacterial species [8]. The biological importance of 6S RNA can be illustrated using the example of the hyperthermophilic bacterium *Aquifex aeolicus* [9] that encodes and expresses a 6S RNA, although it has a parsimonious, highly condensed genome and it abandoned another prominent bacterial ncRNA, the catalytic RNA of RNase P [10]. Phenotypic analyses of 6S RNA-deficient bacterial strains revealed dysregulations under various stress conditions (reviewed in [5,7,11]).

The binding specificity of 6S RNAs for RNAP is achieved by structurally mimicking an open DNA promoter [1,2]. A cryo-EM structure of *E. coli* σ^70^-RNAP in complex with a 6S RNA derivative revealed B-DNA-like helical parameters in the paired regions of 6S RNA as a major component of this structural mimicry [14]. Based on the cryo-EM structure and biochemical/mutational studies in the *E. coli* system, a ‘−35′-like region in the internal (apical) arm of 6S RNA and a ‘−10′-like region located in the 3′-central bubble (3′-CB; see Figure S1A) were pinpointed as the key recognition elements for binding to σ^70^-RNAP [14,15,16].

The open DNA promoter mimicry of 6S RNAs also enables RNAP to utilize the RNA as a template for the transcription of short abortive transcripts termed product RNAs (pRNAs; [17]). The abundance and the length pattern of pRNAs increases with intracellular NTP concentration, particularly of the initiating nucleotide (GTP) in the case of *B. subtilis* 6S-1 RNA-derived pRNA synthesis [12,17,18,19,20]. These pRNAs, when reaching a certain length (13 nt for *E. coli* 6S RNA, 14 nt for *B. subtilis* 6S-1 RNA; [16,18]) and depending on their GC content, have such low rate constants for dissociation from 6S RNA that they stably rearrange the 6S RNA structure. This triggers the release of 6S RNA:pRNA complexes from RNAP as an escape mechanism from the transcriptional block [12,17,21]. In the case of canonical 6S RNAs, this release mechanism is operational during outgrowth of cells from stationary phase upon nutrient resupply [5,12]. The pRNA-induced 6S RNA rearrangement involves the unwinding of 6S RNA helix P2 (termed downstream duplex or helix in the *E. coli* system [14,16]) and the formation of the 6S RNA:pRNA hybrid helix involving nucleotides of the 5′-central bubble (5′-CB) and the 5′-strand of the unwound P2/downstream helix (Figure 1 and Figure S1). For 6S RNAs from *E. coli* and other γ-proteobacteria, the unwinding of the downstream helix allows formation of a 9-bp long hairpin in the 3′-CB (Figure S1A) that was shown to make a major contribution to 6S RNA release induced by pRNAs as short as 13-mers [16]. In contrast, other 6S RNAs, such as *A. aeolicus* 6S RNA or *B. subtilis* 6S-1 RNA, do not form such an extended hairpin in the 3′-CB. In the case of *B. subtilis* 6S-1 RNA, a smaller 3′-CB hairpin already forms in free 6S-1 RNA, which appeared to be stabilized in the 6S-1 RNA:pRNA hybrid structure [12]. However, in *B. subtilis* 6S-1 RNA and *A. aeolicus* 6S RNA, another structural element was inferred to form upon pRNA transcription, termed the ‘central bubble collapse helix (CBCH)’, involving the 3′-strand of P2 and the distal sequence of the 5′-CB (Figure 1; [12,22]. These findings suggest differences in the pRNA-induced 6S RNA release mechanism of *E. coli-* and *B. subtilis*-type 6S RNAs.

Here we show that truncated variants of 6S-1 RNA, solely consisting of the CB flanked by two short helical arms, can still traverse the mechanistic 6S RNA cycle in vitro, despite decreased σ^A^-RNAP affinity. This indicates that the ‘−35′ region is not strictly essential for the basic 6S-1 RNA function. Using strategic 6S-1 RNA mutants, we further characterized the role of the CBCH in the kinetics of the 6S-1 RNA rearrangement/release mechanism and its influence on pRNA length patterns. We observed that certain mutations in the 5′-CB upstream of the pRNA transcription start site (TSS; nt 40) rapidly impair 6S-1 RNA: σ^A^-RNAP ground state binding. Stabilizing the CBCH shifted the pRNA length pattern to shorter pRNAs. A 6S-1 RNA variant with a weakened helix P2 was more effectively refolded and released from the enzyme in the presence of the stabilized CBCH. Our mutational analysis also revealed that formation of a second short hairpin in the 3′-CB is detrimental to 6S-1 RNA release. From our results we conclude that formation of the CBCH supports pRNA-induced 6S-1 RNA refolding and release. We further studied the 6S-1 RNA rearrangement by native PAGE upon stable annealing of LNA versions of pRNAs of different length (6-, 7- and 8-mers) to determine if the rearrangement of 6S-1 RNA structure and 6S-1 RNA:σ^A^-RNAP complex decay may require a certain minimum pRNA length. Finally, we analyzed the shape of free 6S-1 RNA versus 6S-1:pLNA complexes by atomic force microscopy, revealing that 6S-1:pRNA hybrid structures, on average, adopt a more compact structure than 6S-1 RNA alone.

## 2. Materials and Methods

### 2.1. Purification of B. subtilis RNA Polymerase

Native *B. subtilis* σ^A^-RNA polymerase holoenzyme was prepared according to [23] and His-tagged σ^A^-RNAP as described recently [24] (Table 1). The σ^A^-RNAP concentrations were determined by Bradford assay. A calibration curve was established using quick start bovine serum albumin (BSA) (Cat #5000206, Bio-Rad, Feldkirchen, Germany) to prepare 800 µL samples with 0, 2, 4, 6, 8, or 10 µg BSA (referred to the final volume of 1 mL). These samples were mixed with 200 µL of Protein Assay Dye Reagent Concentrate (Cat # 5000006, Bio-Rad) and incubated for 5 min at room temperature. Samples were then transferred into 1 mL cuvettes and absorption was measured at 595 nm in a spectrophotometer. For measurement of test samples, 2 µL of the RNAP preparation was diluted with 798 µL ddH_2_O; all of the following steps were as for the calibration curve samples. For converting weight concentration into molar concentration, we assumed an average molecular weight of 440 kDa for σ^A^-RNAP preparations based on ~340 kDa for the core enzyme plus 60 kDa for sub-stoichiometric amounts of σ^A^, δ and HelD [24].

### 2.2. Cloning of 6S-1 RNA Constructs

The pUC18 derivative plasmid pBB1, encoding the full-length mature 6S-1 RNA (gene: bsrA, 190 nt; [12]) under control of a class III T7 promoter, was chosen as the backbone to construct all 6S-1 RNA mutants (see Table 1). Mutagenesis was performed according to standard procedures. Briefly, the entire plasmid was amplified by PCR, using back-to-back primers (manually designed or using Snapgene 4.1.9) and one mutagenic primer with 5’-proximal or internal mismatches to introduce nucleotide substitutions (see NEB web page, https://international.neb.com/applications/cloning-and-synthetic-biology/site-directed-mutagensis accessed on 13 February 2022). After agarose gel purification of the PCR product, the 5’-ends of primer elongation products were phosphorylated using T4 polynucleotide kinase, the template strands were digested with DpnI and the amplified strands were circularized by T4 DNA ligase in a one-tube reaction. For some constructions, primers were already 5’-phosphorylated before the PCR reaction. After transformation into *E. coli* DH5α cells and selection on ampicillin-containing agar plates, clones were identified by DNA sequencing. For the construction of 6S-1 RNA genes with combined mutations in different regions, the construction was performed in two steps. As an example, the 5’-portion of 6S-1 RNA helix P2 was mutated in the first step as just described. After mutation and verification by DNA sequencing, the plasmid obtained after this first PCR mutagenesis round was used as a template to introduce the compensatory mutations into the 3’-strand of helix P2 in a second PCR. 

### 2.3. In Vitro Transcription of B. subtilis 6S-1 RNA Variants and Synthetic pRNA/pLNA Oligonucleotides

All 6S-1 RNA variants were transcribed from linearized plasmid templates by runoff transcription using T7 RNA polymerase as described, followed by transcript purification usually on 7.5% denaturing (8 M urea) polyacrylamide gels as described [24]. The synthetic 6S-1 pRNA 8-mer (5′-OH-GUU CGG UC) and 14-mer (5′-OH-GUU CGG UCA AAA CU) used as size markers were synthesized by Noxxon Pharma AG (Berlin, Germany) or Integrated DNA Technologies (IDT) Europe. The all-LNA versions of the 8-mer (5′-GTT CGG TC) and 14-mer (5′-GTT CGG TCA AAA CT) were obtained from Axolabs (Kulmbach, Germany).

### 2.4. 6S RNA Refolding

**Figure 2:**
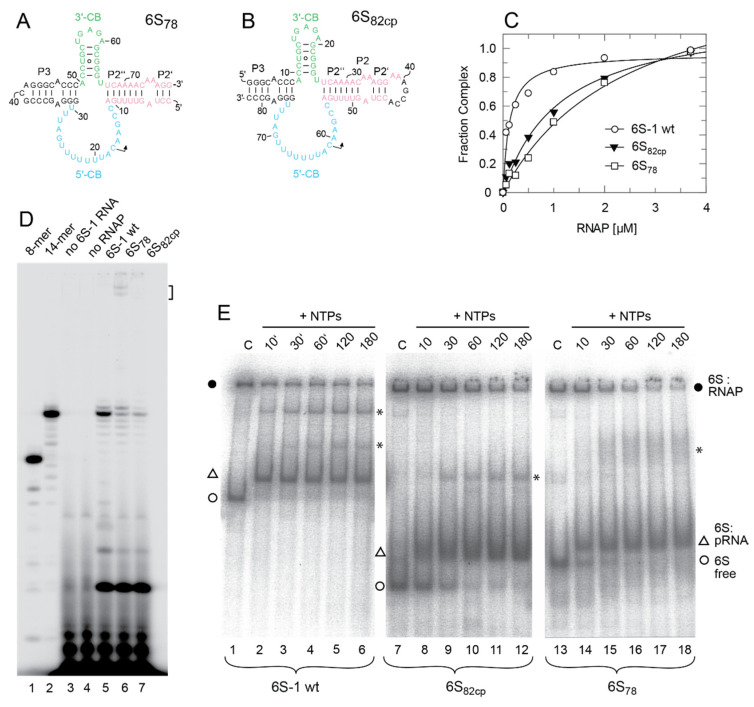
(**A**,**B**) Secondary structures of the truncated 6S-1 RNA derivatives (**A**) 6S_78_ (78 nt) and (**B**) 6S_82cp_ (82 nt, circularly permuted) lacking larger parts of the terminal and internal stem, including the −35 region; color code as in Figure 1. (**C**) Binding of wt 6S-1 RNA, mutant RNA 6S_82cp_, and mutant RNA 6S_78_ to σ^A^-RNAP, analyzed by gel shift assay (see Materials and Methods). A representative experiment is shown. The curves are fits to a one ligand binding site model; as *K*_d_ values for the two mutant RNAs were calculated with endpoints considerably above 1, they are not given here; *K*_1/2_ values (50% complex relative to the endpoint) were ~100 nM for wt 6S-1 RNA and ~1 µM for the two mutant RNAs. (**D**) pRNA transcription using wt 6S-1 RNA or one of the two truncation variants as template (lanes 5–7). Lanes 1 and 2, 5’-^32^P-end-labeled synthetic 6S-1 pRNA 8-mer (lane 1) and 14-mer (lane 2); lanes 3 and 4, controls lacking either 6S RNA (lane 3) or enzyme (lane 4); lanes 5–7: pRNA transcription for 1 h at 37 °C in the presence of 2 µM of the respective 6S RNA variant, 2.5 µM σ^A^-RNAP, 200 µM each NTP, and 250,000 c.p.m of [α-^32^P]-UTP per lane. The bracket at the upper right marks bands attributable to hybrids of 6S_78_ RNA and pRNA runoff transcripts (17-mers). (**E**) Refolded 5’-^32^P-end-labeled wt 6S-1 RNA, 6S_82cp_ or 6S_78_ RNA was preincubated with σ^A^-RNAP for 30 min at 37 °C. Then, NTPs were added to the mixture to induce transcription of pRNAs and the samples were incubated at 37 °C for the time period indicated above each lane. The final concentrations were 1 µM for the 6S-1 RNA variants, ~2 µM for σ^A^-RNAP, and 200 µM each NTP. Lanes C, no NTPs added and incubation at 37 °C for 180 min. Samples for all three 6S RNA variants were analyzed on the same 7.5% native PAA gel; for further details, see Materials and Methods. These experiments were conducted with the native σ^A^-RNAP. Filled circles indicate 6S RNA:RNAP complexes, open triangles 6S RNA:pRNA complexes and open circles free 6S RNA, either wt 6S-1 RNA, 6S_82cp_ or 6S_78_ RNA; asterisks indicate bands of unknown nature not observed in other experiments.

To ensure uniform folding of 6S RNA after denaturing gel purification, 100 nM of T7-transcribed RNA, containing trace amounts of the same 5′-^32^P-labeled RNA (2500 Cherenkov c.p.m. per gel lane), were adjusted to 1 *×* TE buffer (10 mM Tris pH 8.0, 1 mM EDTA). The mixture was then heated to 80 °C in a thermocycler (Biometra, Analytik Jena, Jena, Germany); after 2 min of holding time at 80 °C, the temperature was lowered to 70 °C and kept for 2 min at 70 °C; this was repeated for temperature shifts to 60 and 50 °C, followed by shifting to and maintaining the temperature at 37 °C. For Figure 2E,F, a somewhat different refolding protocol was used: 5 min each at 95, 90, 80, 70, 60, and 50 °C, followed by transfer to 37 °C (≥5 min).

### 2.5. Gel Shift Assay to Assess 6S RNA:σ^A^-RNAP Complex Formation

The following ingredients were combined in a final volume of 10 µL: 1 µL of T7-transcribed and refolded 6S RNA (100 nM) containing trace amounts of the same 5′-^32^P-labeled RNA (2500 Cherenkov c.p.m. per gel lane), 2 µL 5 *×* transcription buffer (200 mM of Tris-HCl pH 8.0, 25 mM MgCl_2_, 800 mM KCl, 5 mM dithiothreitol), 2 µL of heparin solution (400 ng/µL; Sigma-Aldrich, Taufkirchen, Germany), a varying concentration of native or His-tagged σ^A^-RNAP (specified in the corresponding figure legend), and RNase-free ddH_2_O. Dilutions of σ^A^-RNAP stock solutions were made in a RNA polymerase storage buffer [10 mM Tris-HCl pH 8.0, 10 mM MgCl_2_, 0.1 mM EDTA, 0.1 mM dithiothreitol, 0.1 M NaCl and 50% (*v*/*v*) glycerol]. The aforementioned 10 µL reaction mixtures were incubated for 30 min at 37 °C, followed by mixing with 10 µL 2 *×* native loading dye [10% (*v*/*v*) glycerol, 10 mM MgCl_2_, 0.025% (*w*/*v*) bromophenol blue and 0.025% (*w*/*v*) xylene cyanol blue)] and loading onto a native 7.5% native polyacrylamide (PAA) gel [running buffer 1 *×* TBE; glass plate dimensions 30 cm *×* 20 cm *×* 1 mm (L *×* B *×* H)], typically run for 3 h at 25 mA. In the experiments shown in Figure 2C, native PAGE was conducted in an electrophoresis buffer containing 0.5 *×* TBE, 160 mM KOAc, 5 mM Mg(OAc)_2_, 1 mM DTT, pH 8.6, with correspondingly adjusted electric field strength to prevent substantial warming of the gel during electrophoresis [12]. Gels were exposed overnight to a phosphor imaging plate (Fujifilm, Düsseldorf, Germany). Radioactive bands were visualized and quantified using a FLA-3000 Fluorescent Image Analyzer (Fujifilm), the software BAS Reader (version 3.14), and AIDA (v. 3.45.039). Data fitting of binding curves was based on three independent experiments (if not stated otherwise), using the software Grafit, v. 5.0.13 (Erithacus Software, East Grinstead, West Sussex, UK).

### 2.6. Native Gel Assay to Assess NTP Dependence of the pRNA-Induced Rearrangement of 6S-1 RNA and Its Release from RNAP

For the NTP concentration variation experiment in Figure 5, a set of 10.5 µL mixtures containing 3.5 µL 6S-1 RNA (100 nM stock concentration with trace amounts of 5′-^32^P-labeled 6S-1 RNA; a total of ~9000 Cherenkov c.p.m. for withdrawal of 3 time point aliquots, corresponding to 2500 c.p.m. per gel lane; see below) in 1 *×* TE buffer were subjected to the refolding protocol. Then, 7 µL of 5 *×* transcription buffer (see above), 7 µL of heparin solution (400 ng/µL), and ~1 µL of ddH_2_O were added per reaction. To each sample, with a time interval of 7 min, 2.5 µL of native σ^A^-RNAP were added (resulting in a volume of 28 µL). The 7-min time delay was required for staggered processing of samples with different NTP concentrations. Each sample was incubated for 30 min at 37 °C to reach equilibrium of 6S-1 RNA binding to σ^A^-RNAP. Reactions were started by adding 7 µL of NTP mix (final reaction volume: 35 µL) to adjust the NTP concentration to either 40, 80, 120, 160, or 200 µM each NTP [A/C/G/UTP mixes of 200 µM, 400 µM, 600 µM, 800 µM and 1000 µM of each NTP were usually made from 100 mM stock solutions (Carl Roth, Karlsruhe, Germany) by dilution in ddH_2_O]; the final concentration of 6S-1 RNA was 10 nM and that of σ^A^-RNAP was 2 µM. After 15 s, 1 min, and 2 min at 37 °C, 10 µL aliquots were withdrawn, mixed with 10 μL of 2 *×* native loading dye (see above), and put immediately on ice. The three collected aliquots were then loaded onto a 7.5% native PAA gel [running buffer 1 *×* TBE; glass plate dimensions 30 cm *×* 20 cm *×* 1 mm (L *×* B *×* H)] that was running at low current (~5 to 10 mA) to avoid diffusion of initially loaded samples from gel pockets until all pockets were loaded. After loading the last aliquot set, the current was increased to 25 mA for 3 h. Phosphor imaging and data evaluation were performed as described in Section 2.5.

### 2.7. Native Gel Assay to Analyze 6S-1 RNA Rearrangement Kinetics at a Specific NTP Concentration

A total of 5.5 µL of 5′-^32^P-labeled 6S RNA (~2500 Cherenkov c.p.m per µL) was combined with 5.5 µL of 2 × TE buffer (20 mM Tris pH 8.0, 2 mM EDTA) and refolded as described in Section 2.4. Then, 11 µL of 5 × transcription buffer (see above), 11 µL of heparin solution (400 ng/µL), between ~12 and 19 µL of His-tagged σ^A^-RNAP (stock concentrations varied between 5.75 and 9.05 µM), and RNase-free ddH_2_O were added to a total volume of 53.6 µL. The reaction mixture was incubated for 1 h at 37 °C to equilibrate 6S RNA binding to σ^A^-RNAP. Subsequently, 1.4 µL of an A/C/G/UTP mix (2 mM each NTP) was added (final volume: 55 µL) to start the reaction at 37 °C. The reaction mixtures contained final concentrations of usually 2 µM (2.6 µM in Figure 10D) His-tagged σ^A^-RNAP, 50 µM each NTP, and trace amounts (2500 Cherenkov c.p.m per gel lane) of 5′-^32^P-labeled 6S RNA. ATP omission experiments and the experiment shown in Figure 6A followed the same protocol, except that the concentration of each added NTP was 100 µM instead of 50 µM. Deviations from this protocol are specified in the legend to Figure 2E. Typically, 10 µL aliquots were withdrawn after 15 s and after 2, 5, 10, and 15 min, to which 10 µL of 2 × native loading dye (see above) were added, followed by shock freezing in liquid N_2_. Samples were loaded onto a native 7.5% PAA gel (running buffer 1 × TBE; gel dimensions see above) that was run for 3 h at 25 mA. Control samples lacking NTPs, or enzyme and NTPs, were prepared and handled in the same manner as the test samples before loading onto the gel (incubation time at 37 °C for at least 15 min). Phosphor imaging and data evaluation were performed as described in Section 2.5. After electrophoresis, the band corresponding to the 6S-1 RNA:RNAP complex (signal 1) and the lane segment (signal 2) covering both free 6S-1 RNA and rearranged 6S-1 RNA:pRNA hybrids were quantified for each lane; the sum of signals 1 and 2 yielded the total radioactivity in each lane; the radioactivity of the complex band (signal 1) was then divided by the total radioactivity (signal 1 + 2) in the lane, and the resulting complex fraction was normalized to the fraction of complex at time point zero. Data fitting was based on three independent experiments using the software Grafit, v. 5.0.13.

### 2.8. pRNA Transcription Assay Using Native σ^A^-RNAP

**Figure 3:**
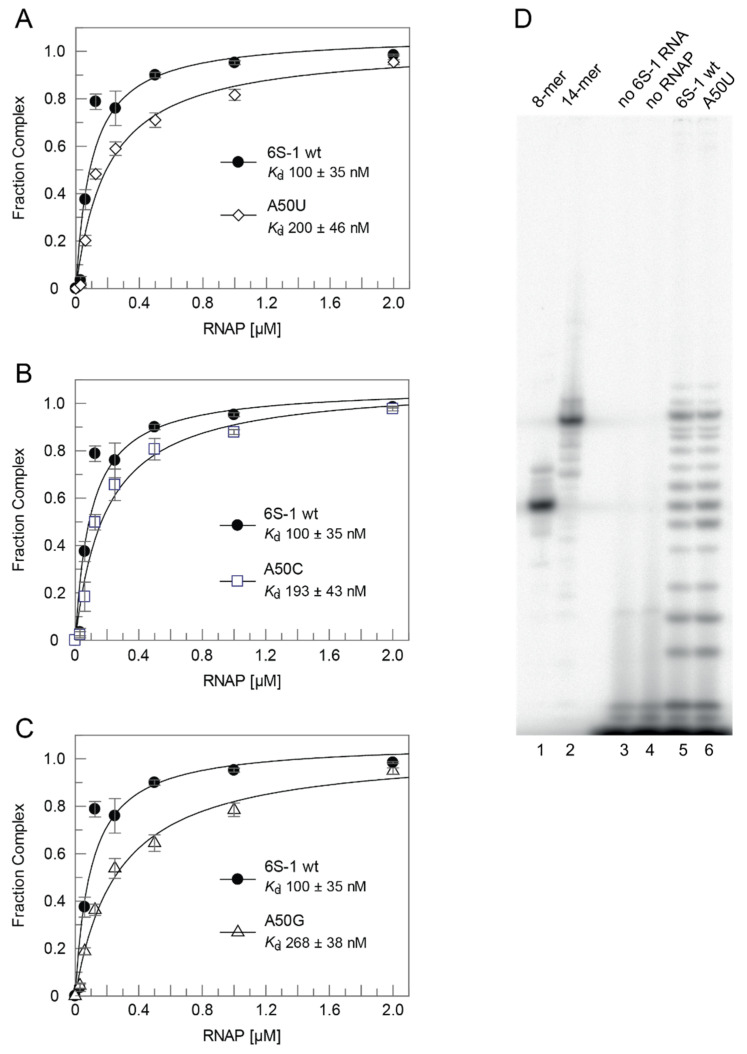
σ^A^-RNAP affinity of 6S-1 RNA A50 mutants and pRNA transcription pattern of the A50U mutant RNA. (**A**–**C**) σ^A^-RNAP affinity of mutant RNAs (**A**) A50U, (**B**) A50C and (**C**) A50G relative to wt 6S-1 RNA, measured by gel shift assay using the native σ^A^-RNAP. The calculated *K*_d_ values (fit to a one ligand binding site model) are indicated within each graph; data points in graphs A-C were based on three independent experiments each (error bars, SEM). (**D**) pRNA transcription using the wt (lane 5) and A50U mutant (lane 6) 6S-1 RNA as template, performed as in Figure 2D. Lanes 1 and 2, 5’-^32^P-end-labeled synthetic 6S-1 pRNA 8-mer (lane 1) and 14-mer (lane 2); lanes 3 and 4, controls lacking either 6S RNA (lane 3) or enzyme (lane 4). For details, see Materials and Methods. These experiments were conducted with the native σ^A^-RNAP.

**Figure 4:**
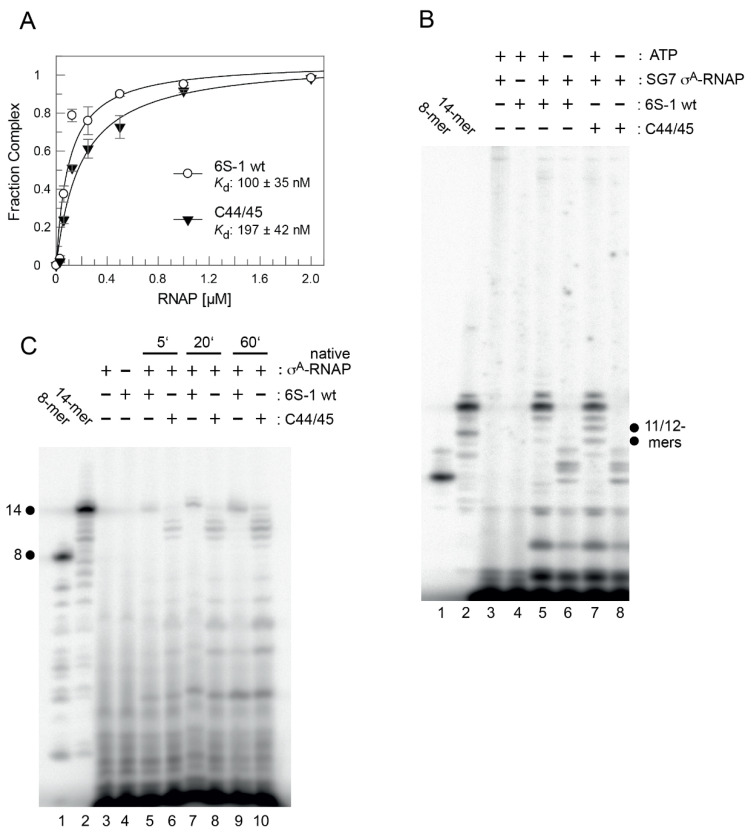
σ^A^-RNAP binding affinity and pRNA transcription pattern for the wt and C44/45 mutant 6S-1 RNAs. (**A**) Binding affinity of mutant C44/45 (see Figure 1) and wt 6S-1 RNA to native σ^A^-RNAP; *K*_d_ values, determined as in Figure 3A–C, are indicated within the graph. (**B**) pRNA transcription for 1 h at 37 °C using the wt (lanes 4–6) or C44/45 mutant (lanes 7 and 8) 6S-1 RNA as template (2 µM), σ^A^-RNAP (0.41 µM) prepared from strain SG7, 270 µM each NTP (lanes 3–5, 7), or 270 µM CTP, UTP and GTP (lanes 6 and 8), and 250,000 c.p.m of [α-^32^P]-UTP per lane. Lanes 1 and 2, 5’-^32^P-end-labeled synthetic 6S-1 pRNA 8-mer (lane 1) and 14-mer (lane 2) used as size markers; lanes 3 and 4, controls lacking 6S RNA (lane 3) or enzyme (lane 4); dots indicate the increased abundance of mainly pRNA 11- and 12-mers in lane 7 vs. 5. The observation of pRNAs longer than 8-mers in the absence of ATP indicates non-templated product extension or misincorporation of NTPs other than ATP. For details, see Materials and Methods. (**C**) pRNA transcription for 1 h 37 °C on wt or C44/45 mutant 6S-1 RNA (2 µM) using the native σ^A^-RNAP (1 µM), 200 µM each NTP, and 250,000 c.p.m of [α-^32^P]-UTP per lane. For more details, see Materials and Methods.

For the experiment shown in Figure 3D, 2 µL of in vitro T7-transcribed 6S RNA (stock concentration: 15 μM) were combined with 2 µL of 2 *×* TE buffer and subjected to the refolding protocol (see above). Then, 3 µL of 5 *×* transcription buffer (see above), 2.4 µL of native σ^A^-RNAP (stock concentration: 15.84 µM), and 3.1 µL of RNase-free ddH_2_O were added to a volume of 12.5 µL. The reaction mixture was incubated for 15 min at 37 °C to equilibrate binding of 6S RNA to σ^A^-RNAP. Subsequently, 2 µL of NTP mix (ATP, GTP, CTP and UTP, each 1.5 mM) plus 0.52 µL [α-^32^P]UTP (250,000 Cherenkov c.p.m per reaction mixture) were added. The final concentrations in the reaction mixture were 2 μM for the 6S RNA variant, 2.5 μM for native σ^A^-RNAP, and 200 μM for each NTP. The final reaction mixture (~15 µL) was incubated for up to 1 h at 37 °C. After incubation, 5 µL of the reaction mixture was withdrawn and mixed with 15 µL of 2 *×* denaturing loading dye [95% (*v*/*v*) formamide, 0.025% (*v*/*v*) SDS, 0.025% (*v*/*v*) bromophenol blue, 0.025% (*v*/*v*) xylene cyanol, 0.5 mM EDTA], heated for 3 min to 98 °C in a dry bath (Biometra, Analytik Jena, Jena, Germany), followed by cooling on ice for 5 min. Control samples (final volume: 15 µL) either lacking native σ^A^-RNAP or without 6S RNA were subjected to the same conditions as the test samples. Samples were loaded onto denaturing 25% PAA gel [8 M urea; running buffer 1 x TBE; gel plate dimension 42 cm *×* 33.5 cm *×* 0.3 cm (L *×* B *×* H)] that was run for 16 h at 1200 V. Phosphor imaging and data evaluation was performed as described in Section 2.5. A slight variation of this protocol was applied to pRNA transcription of RNAs 6S-1 wt and C44_45 (Figure 4C). Here, the differences included a final volume of 20 instead of 15 µL, a native σ^A^-RNAP final concentration of 1 μM, and withdrawal of 5 µL aliquots at time points 5-, 20-, and 60-min post-nucleotide addition.

### 2.9. pRNA Transcription Assay Using His-Tagged σ^A^-RNAP

4 µL of T7-transcribed 6S RNA (stock concentration ~15 μM) and 4 µL of 2 × TE buffer (20 mM Tris pH 8.0, 2 mM EDTA) were mixed and subjected to the RNA refolding procedure (see above). Then, 6 µL of 5 × transcription buffer (see above), ~1 to 5 µL of His-tagged σ^A^-RNAP (stock concentration varied from 5.75 µM to 9.05 µM), and RNase-free ddH_2_O were added to a total volume of 24.2 µL, followed by equilibration for 15 min at 37 °C. Approximately 12 µL aliquots of this reaction mixture were transferred into two new tubes, followed by the addition of 2.9 µL of A/C/G/UTP mix or C/G/UTP mix (1.39 mM each NTP); the resulting ~15 µL contained [α-^32^P]UTP (250,000 Cherenkov c.p.m per lane), 270 µM end concentration of each indicated NTP, 2 μM 6S RNA, and ~0.4 µM His-tagged σ^A^-RNAP; pRNA transcription was allowed to proceed for 1 h at 37 °C. Thereafter, 5 µL of the reaction mixture was withdrawn and mixed with 15 µL of 2 × denaturing loading dye (see above), heated for 3 min to 98 °C in a dry bath, followed by cooling on ice for 5 min. Control samples either lacking His-tagged σ^A^-RNAP or 6S RNA were treated in the same manner. For details on electrophoresis, see the preceding paragraph. 

### 2.10. Annealing of pLNA/pRNA Oligonucleotides to 6S-1 RNA

A total of 10 pmol of 6S-1 RNA were mixed with trace amounts of 5’-^32^P-labeled 6S-1 RNA (10,000 Cherenkov c.p.m.) and 0.4 µL 10 × TE buffer. The volume was adjusted to 4 µL with RNase-free ddH_2_O. Then, 2 µL of a 50 µM stock solution of the respective pLNA or pRNA oligonucleotide was added and the mixture was successively incubated for 5 min each at 95, 90, 80, 70, 60, and 50 °C, followed by transfer to 37 °C for at least 5 min. Subsequently, 1 µL of heparin (400 ng/µL), 2 µL of 5× transcription buffer (see above), and either ~1.1 µL native σ^A^-RNAP (stock concentration: 8 mg/mL) or 1.1 µL double-distilled RNase-free ddH_2_O (for controls without RNAP) were added. Mixtures were incubated for 30 min at 37 °C, then supplemented with 10 µL of 2 × native RNA loading dye (see above) and analyzed by 10% native PAGE.

### 2.11. Atomic Force Microscopy (AFM)

For AFM analysis, the RNA was prepared as follows: 1 µL of a 10 µM stock solution of in vitro T7-transcribed 6S-1 RNA was mixed with a tenfold molar excess of the respective pLNA oligonucleotide in a final volume of 10 µL adjusted to 1 *×* TE buffer. The annealing protocol was the same as described in the preceding paragraph. To remove excess pLNA oligonucleotides, samples containing the pLNA 8- or 14-mer were centrifuged 3 times, with 500 µL 1× TE buffer per round, through Amicon Ultra centrifugal filter units (molecular weight cut-off: 10 kDa). Before the actual measurements, the density of molecules was recorded in a test image; depending on the observed density, RNA mixtures were diluted with 1 × TE buffer to adjust the working concentrations to ~1–4 nM. RNA solutions were transferred to a mica surface treated with poly-L-lysine (MW 500–2000, Sigma-Aldrich, Taufkirchen, Germany) as described [26]. Pictures were taken using a Veeco Multimode Nanoscope IIIa AFM device with silicon tips (Tap300Al-G, BudgetSensors, Sofia, Bulgaria) in tapping mode (resonance frequency: 300 kHz, scan rate: 1 Hz). Pictures were recorded with an edge length of 1 µm. The AFM images were analyzed using ‘ImageJ 1.51’ [27]. Particles with a size of 0.3 to 0.64 µm^2^ and a circularity (c = 4π × [area]/[perimeter]^2^) of 0.2 to 0.6 were annotated as 6S-1 RNA molecules. For each of these particles, an ellipse was fitted. The ratio of the longitudinal (long) and latitudinal (short) axis length (see Figure 13C) were used to describe the molecule shape. Smaller ratios are indicative of more compact and possibly bent molecules while larger ratios indicate more stretched conformations. 

### 2.12. RNAfold and RNAcomposer Predictions

Secondary structure predictions were computed on the RNAfold web server 2.4.18 ([28]; http://rna.tbi.univie.ac.at, accessed on 23 December 2021) using the default parameters. For simulating complexes of 6S-1 RNA and pRNAs, the corresponding pRNA binding site was blocked using constraint folding with the Enforce Constrained pairing pattern option. The 3D structure predictions were performed with RNAcomposer ([29]; https://rnacomposer.cs.put.poznan.pl, accessed on 15 December 2021). For RNAcomposer prediction of the 6S-1:pRNA complex, the 6S-1 RNA sequence was extended at the 5’-end by a linker sequence (here 25 cytidines) and the pRNA sequence at the very 5’-end, mimicking an artificial pseudoknot structure; very similar results were obtained with U25, A25, and G25 linkers or longer homopolymeric linkers of 50, 75, or 100 nt that were analyzed in comparison.

## 3. Results

### 3.1. Analysis of 6S-1 RNA Derivatives with Large Truncations

In the *E. coli* system, biochemical studies and the cryo-EM structure of the σ^70^-RNAP complex have led to the identification of structural 6S RNA elements that correspond to the −35 and −10 regions of DNA promoters and interact with σ^70^ as well as the β and β’ subunits of the core RNAP [14,15,16]. These 6S RNA elements coincide with the 3’-CB (−10 element) and, by inference from the *E. coli* system, roughly with the P5 helix and its two flanking internal loops (−35 element) in *B. subtilis* 6S-1 RNA (Figure 1). The P2 region interacts with β and β’, while the terminal stem region (corresponding to helix P1 in Figure 1A) was shown to be dispensable for complex formation [14,15]. To gain insight into the interaction of *B. subtilis* 6S-1 RNA and σ^A^-RNAP, we constructed two extensively truncated derivatives of *B. subtilis* 6S-1 RNA, a 78-nt long variant (6S_78_) and a very similar but circularly permuted variant (6S_82cp_) that was 82-nt in length, both lacking the ‘−35’ region (Figure 2A,B). The 6S_82cp_ variant was constructed to evaluate possible effects caused by fraying of the 5’- and 3’-termini upon helix P2 disruption during pRNA synthesis. Both truncation variants showed 50% complex formation with σ^A^-RNAP at ~1 µM enzyme, whereas the same enzyme bound full-length 6S-1 RNA with a *K*_d_ of ~100 nM (Figure 2C). Nevertheless, the truncated RNAs preserved the ability to serve as a template for the synthesis of pRNA 14/15-mers (Figure 2D, lanes 6 and 7), although with reduced efficiency relative to full-length 6S-1 RNA (lane 5). Particularly low pRNA synthesis efficiency in the case of RNA 6S_82cp_ can be attributed to the constraints imposed by the artificial capping loop of helix P2. RNAs 6S_78_ and 6S_82cp_ were converted to complexes with pRNA upon incubation with NTPs but not as efficiently as the full-length (wt) 6S-1 RNA (Figure 2E). Likewise, both truncated 6S RNAs showed the hallmark feature of time-dependent release from RNAP upon induction of pRNA synthesis (Figure 2E). Altogether, these findings demonstrate that the basic mechanistic features of 6S-1 RNA are maintained, though with reduced efficiency, when the RNA’s size is reduced to the CB solely flanked by the immediate helical elements that confine the CB. At the same time, the results demonstrate the contribution of structural elements in the helical arms of native 6S-1 RNA, such as the putative −35 region, to ground state binding affinity of 6S-1 RNA for σ^A^-RNAP.

### 3.2. Role of the Central Bubble Collapse Helix (CBCH) in the Rearrangement and 6S-1 RNA Release from RNAP

The rearrangement and release mechanism of *B. subtilis* 6S-1 RNA differs from that of 6S RNAs of *E. coli* and related γ-proteobacteria. In the latter, the 3’-CB is essentially unstructured in the free state, but an extended hairpin (9 bp) forms between nucleotides of the 3’-CB and the 3’-strand of helix P2 (also called downstream duplex; [14]), when P2 is disrupted during pRNA synthesis. This hairpin was shown to play a key role in the release mechanism in the *E. coli* system [16]. As a shorter hairpin (with 5 and potentially up to 7 bp according to RNAfold prediction; Figure 1A) already forms in the 3’-CB of free *B. subtilis* 6S-1 RNA [12] and no extended hairpin forms during pRNA synthesis, this raised the question of whether the CBCH may play a supporting role in the 6S-1 RNA release process in addition to formation of the pRNA:6S-1 RNA hybrid helix. We initially approached this question by introducing the A50U mutation into 6S-1 RNA, which was expected to slightly stabilize the CBCH (Figure 1B). When we tested this mutant 6S-1 RNA for binding to σ^A^-RNAP, we observed a two-fold increase in *K*_d_ (Figure 3A). Similar two- to threefold *K*_d_ increases were also observed for A50C and A50G mutations (Figure 3B,C), indicating that the base identity of residue A50 in the 5’-CB plays a role in the interaction with σ^A^-RNAP. We then tested if the CBCH stabilization by the A50U mutation may energetically favor the pRNA-induced rearrangement of 6S-1 RNA and thereby somewhat shift the pRNA length pattern to shorter pRNAs. However, the pRNA length pattern did not differ substantially from that of wt 6S-1 RNA (Figure 3D). For a more substantial stabilization of the CBCH, we mutated residues U44/45 to cytosines (termed mutant C44/45 in the following), thus converting the tandem G:U to G:C pairs (Figure 1B). This double mutation decreased σ^A^-RNAP binding affinity by a factor of two (Figure 4A) and up to a factor of three with other RNAP preparations (not shown). Here, pRNA transcription assays clearly revealed a higher proportion of pRNAs shorter than 14 nt (mainly 11- and 12-mers) when using the SG7 RNAP as enzyme and the C44/45 mutant RNA as a template (Figure 4B, lane 7 vs. 5, marked at the right margin). This effect was even enhanced (for unknown reasons) with the native RNAP (Figure 4C). These findings provided evidence that the C44/45 double mutation may energetically favor the pRNA-induced rearrangement of 6S-1 RNA to such an extent that pRNAs shorter than 14 nt (10 to 12-mers) can now more effectively trigger the rearrangement of 6S-1 RNA and its release from σ^A^-RNAP.

**Figure 5:**
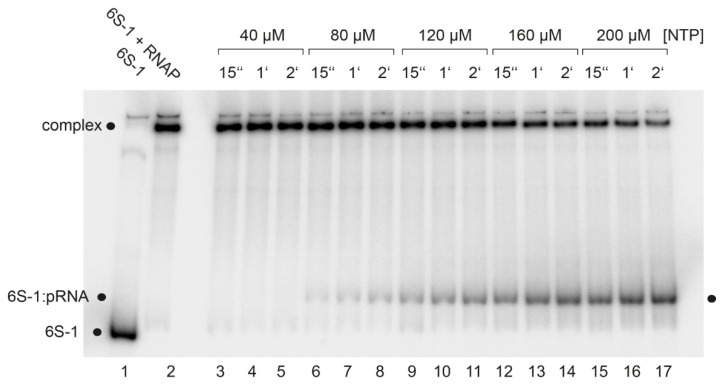
NTP concentration dependence of pRNA synthesis as well as 6S-1 RNA rearrangement and release, analyzed by gel shift assay (7.5% native PAGE). Lane 1, 6S-1 RNA (10 nM 6S-1 RNA); lane 2, as lane 1 plus 2 µM native σ^A^-RNAP; lanes 3–17, as lane 2, but followed by adjustment to the indicated NTP concentration and withdrawal of aliquots at 15 s, 1 min, and 2 min post-NTP addition. For further details, see Materials and Methods.

**Figure 6:**
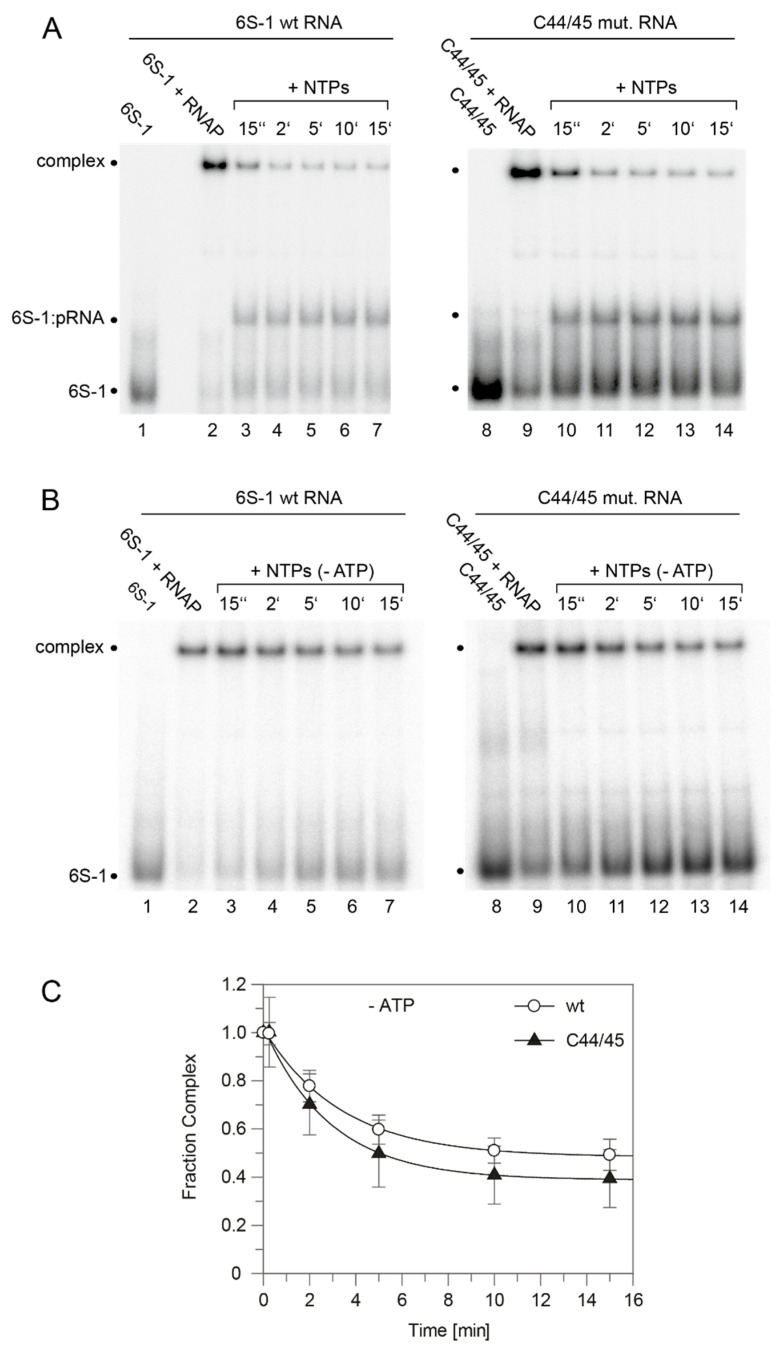
pRNA-induced rearrangement and release of wt and C44/45 mutant 6S-1 RNA upon addition of (**A**) all four NTPs or (**B**) CTP/GTP/UTP only. Each NTP had a final concentration of 100 µM. (**A**) Lanes 1 and 8, 5′-^32^P-labeled 6S-1 RNA only; lanes 2 and 9, 6S-1 RNA incubated with 2 µM SG7 σ^A^-RNAP; lanes 3–7 and 10–14, as lanes 2 and 9, but followed by addition of NTPs and withdrawal of aliquots at the indicated time points. (**B**) As in panel A, but addition of CTP/GTP/UTP only (-ATP) in lanes 3–7 and 10–14. (**C**) Kinetics of wt and C44/45 6S-1 RNA release from σ^A^-RNAP upon pRNA-induced 6S-1 RNA refolding in the presence of CTP/GTP/UTP only; based on two independent experiments including the one shown in panel B (error bars, SEM). The data was fit to an equation for a single exponential with offset, yielding rate constants *k* of 0.3 ± 0.03 min^−1^ (wt) and 0.35 ± 0.04 min^−1^ (C44/45) and offsets (= endpoints representing release-resistant complexes) of 0.48 ± 0.01 (wt) and 0.39 ± 0.02 min^−1^ (C44/45). For further details, see Section 2.7.

**Figure 7:**
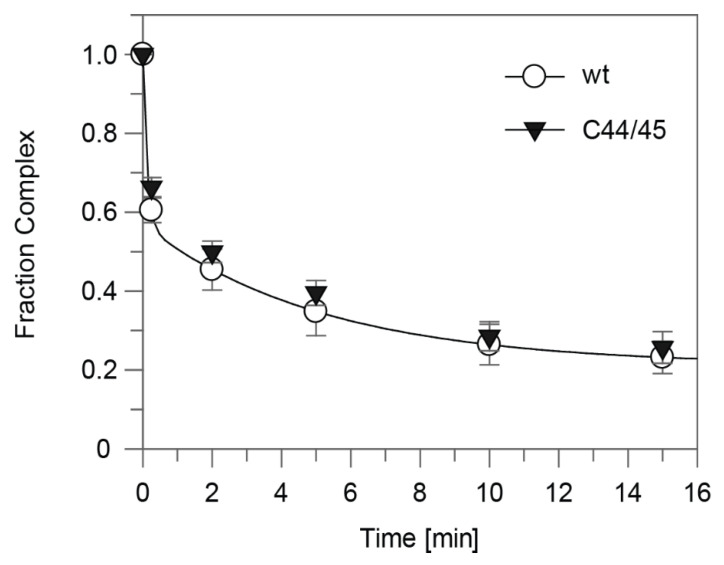
Kinetics of wt and C44/45 6S-1 RNA release from σ^A^-RNAP (= complex decay) upon pRNA-induced 6S-1 RNA refolding. The setup was the same as in Figure 6A but conducted with only 50 µM of each NTP. The data was fit to an equation for a double exponential curve with offset. The rate constant *k*_1_ for the fast phase was calculated as 8.4 ± 0.02 min^−1^ (wt) and 7.2 ± 1.6 min^−1^ (C44/45) that for the slow phase (*k*_2_) as 0.19 min^−1^ (wt) and 0.17 ± 0.04 min^−1^ (C44/45); the release-resistant complex fraction (at the endpoint) was 0.21 (wt) and 0.22 (C44/45). For details, see Section 2.7.

As a next step, we evaluated the 6S-1 RNA rearrangement/release kinetics as a function of NTP concentration (Figure 5). In this setup, complexes of 6S-1 RNA and σ^A^-RNAP were preformed and pRNA transcription was induced by the addition of NTP substrates, followed by withdrawal of aliquots at different time points. The experiment illustrated in Figure 5 showed that the pRNA-mediated rearrangement of 6S-1 RNA (resulting in retarded gel mobility) and its release from σ^A^-RNAP gains substantial momentum at NTP concentrations >40 µM. Evidently, there are two subpopulations of 6S-1 RNA:σ^A^-RNAP complexes, one reacting in the fast phase of the reaction (≤15 s) and the other reacting with slower kinetics (see also Figure 7). The subpopulation reacting in the fast phase increases with increasing NTP concentration (Figure 5). We then analyzed the rearrangement kinetics for wt 6S-1 RNA and the C44/45 mutant RNA at 100 µM each NTP to determine if the C44/45 RNA may be released faster than the wt RNA. Yet, no significant differences between the wt and C44/45 mutant RNA were observed and major fractions of both RNAs were released during the initial fast phase (≤15 s) (Figure 6A; quantification not shown). We also investigated the rearrangement/release kinetics under ATP omission conditions (only CTP/GTP/UTP added, which should restrict pRNA synthesis to 8-mers) to examine if the C44/45 mutant RNA might be more efficient in RNA release from RNAP, as suggested by the pRNA transcription pattern (Figure 4B,C). As expected from previous results [12], stable 6S-1 RNA:pRNA complexes could not be resolved in gel shift assays upon ATP omission (Figure 6B). The decay of 6S-1 RNA:RNAP complexes was very slow and essentially lacked an initial fast phase of release with both RNA variants (Figure 6C), suggesting that fast release requires the synthesis of pRNAs > 8-mers. The C44/45 mutant RNA showed a trend toward somewhat faster release and lower endpoint relative to wt 6S-1 RNA (Figure 6C).

**Figure 8:**
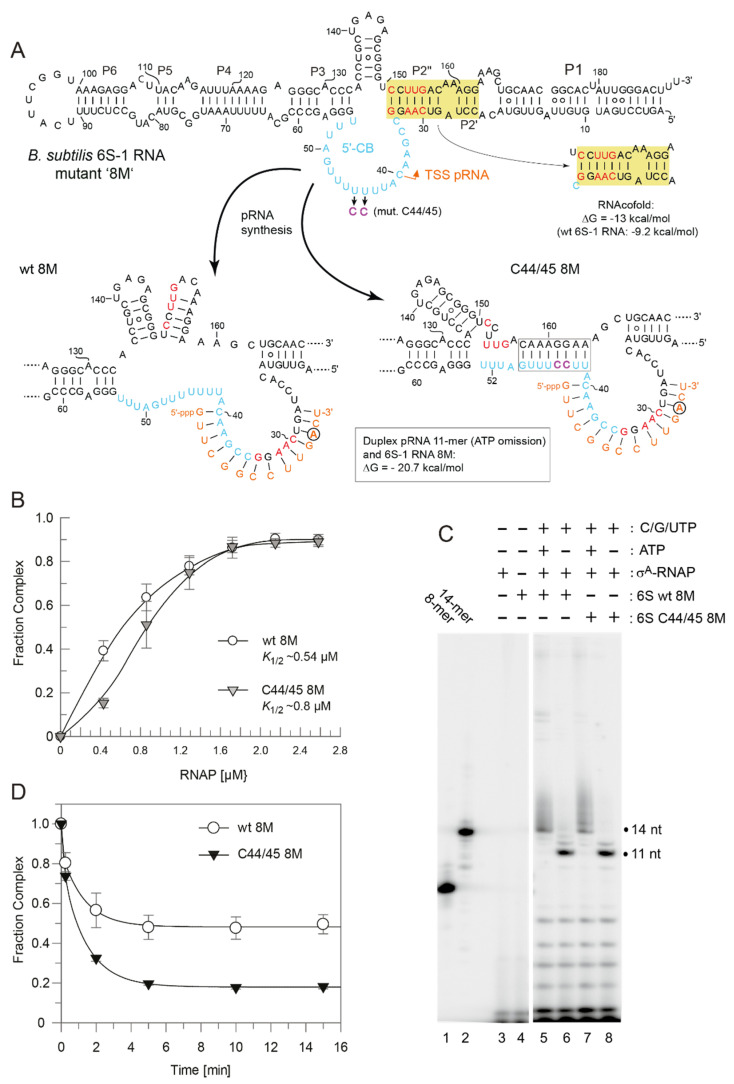
Characterization of wt 8M and C44/45 8M 6S-1 RNAs. (**A**) Secondary structure of the two 6S-1 RNA variants in the free state (top) and after the structural rearrangement (bottom) induced by pRNA transcription. The eight mutations (8M) are highlighted in red, the C44/45 mutations in magenta. In the sketches of the rearranged structures (bottom), the favored structures based on RNAfold dot plot analysis are shown. The first A residue in the pRNA sequence is marked by a circle. The ΔG of the 6S-1 8M RNA:pRNA 11-mer hybrid helix is predicted as −20.7 kcal/mol (RNAfold), compared with −22.4 for a pRNA 14-mer annealed to wt 6S-1 RNA (Figure 1B). (**B**) σ^A^-RNAP binding affinity of the two 6S-1 RNA variants. *K*_1/2_ gives the σ^A^-RNAP concentration at half-maximal saturation. The curves are approximating curves (B-spline of order 3; data point errors, SEM). (**C**) pRNA transcription by SG7 σ^A^-RNAP (0.45 µM) using either wt 8M or C44/45 8M RNA (1.06 µM) as template, and in the presence of all four NTPs (each 200 µM) or with CTP, GTP, and UTP (each 200 µM) but lacking ATP, as indicated above lanes 5–8. Lanes 1 and 2, pRNA 8- and 14-mer markers; lanes 3 and 4, controls lacking either 6S RNA (lane 3) or enzyme (lane 4). These experiments were conducted with SG7 σ^A^-RNAP. (**D**) Kinetics of pRNA-induced complex decay for wt 8M or C44/45 8M RNAs using SG7 σ^A^-RNAP (2 µM) and 100 µM each CTP, GTP and UTP (see Section 2.7 for details); under these conditions, mainly pRNA 11-mers were synthesized based on the results shown in panel C. Data evaluation was performed as described in the legend to Figure 7 and in Section 2.7. The rate constants *k*_1_ for the fast phase could not be stably predicted, those for the slow phase (*k*_2_) were 0.78 ± 0.12 min^−1^ (wt 8M) and 0.72 ± 0.08 min^−1^ (C44/45 8M), the release-resistant complex fractions were 0.48 (wt 8M) and 0.18 (C44/45 8M).

**Figure 9:**
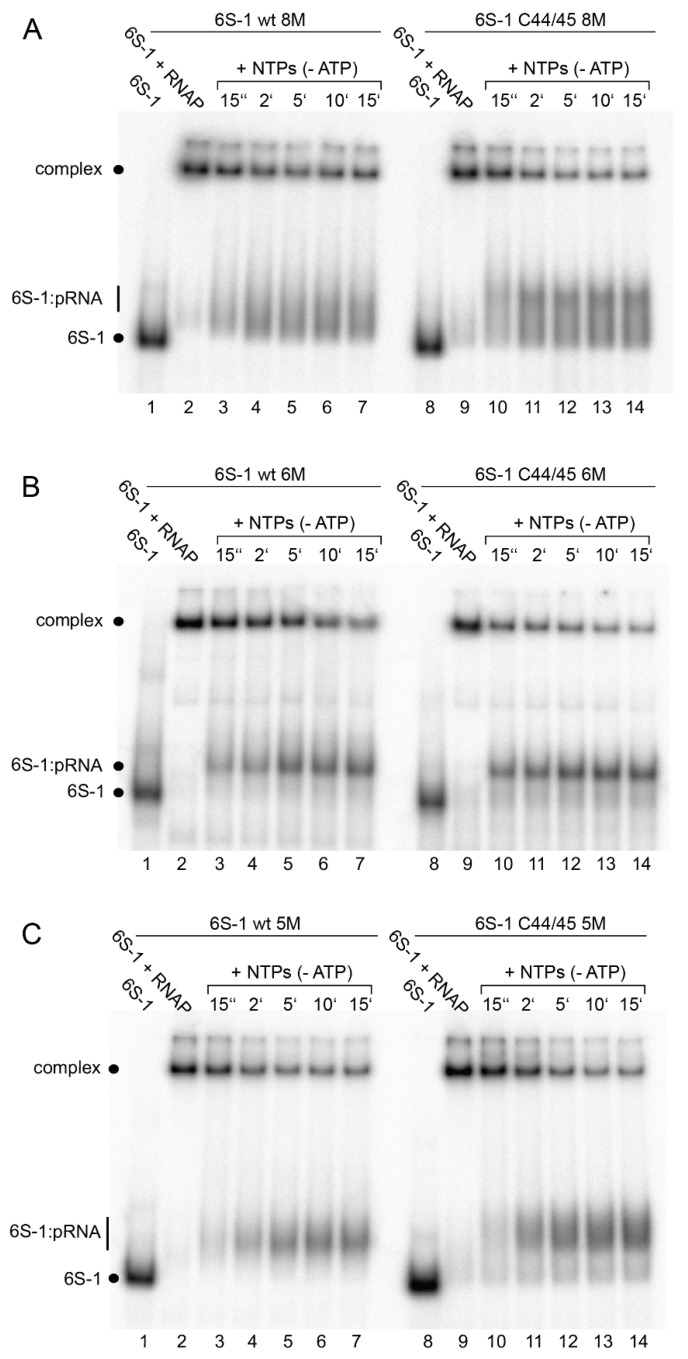
pRNA-induced rearrangement of 6S-1 RNA variants (**A**) wt and C44/45 8M, (**B**) wt and C44/45 6M, and (**C**) wt and C44/45 5M, using 5′-^32^P-end-labeled 6S-1 RNA, 2 µM SG7 σ^A^-RNAP, and 100 µM each CTP, GTP, UTP, analyzed by 7.5% native PAGE. For details, see Section 2.7.

For better handling of manual kinetics, we reduced the concentration for each of the four NTPs from 100 to 50 µM each. Comparisons of the release (complex decay) kinetics of wt and C44/45 mutant 6S-1 RNA yet provided no evidence for differences between the two (Figure 7). To accentuate the differences between wt and C44/45 mutant 6S-1 RNAs, we designed RNA variant 8M (8 mutations in P2) in the wt and C44/45 context (Figure 8A). Beforehand, we observed that major sequence changes in P2 adjacent to the CB (6S-1 RNA mutants UUUUswap and P2swap) had no or very minor effects (<two-fold) on ground state binding (Figure S2). The rationale for constructing the 8M mutant RNA was to change the pRNA-coding sequence such that pRNA 11-mers are transcribed in the absence of ATP (versus 8-mers in the case of wt RNA); the generated 11-mer:6S-1 8M RNA duplexes were predicted to have a ΔG of −20.7 kcal/mol (Figure 8A), thus close to the stability of the duplex formed between wt 6S-1 RNA and complementary pRNA 14-mers (ΔG = −22.4 kcal/mol; Figure 1B). With the 8M design, we speculated we would see a more pronounced advantage of the C44/45 8M vs. wt 8M variant to undergo the structural rearrangement in the presence of such shorter pRNA:6S-1 RNA hybrids. As a side effect, the 8M mutations slightly stabilized helix P2 (predicted ΔG = −13 vs. −9.2 kcal/mol for the wt RNA; Figure 8A). Again, σ^A^-RNAP affinity was ~1.5-fold lower for the C44/45 8M relative to the wt 8M RNA (Figure 8B), but pRNA transcription patterns were essentially identical (Figure 8C). The 8M variants now formed gel-resolvable 6S:pRNA 11-mer complexes under ATP omission conditions (Figure 9A). However, when we analyzed the 6S RNA release kinetics, we observed a much lower fraction of released 6S RNA at the endpoint for variant wt 8M (52%) relative to variant C44/45 8M (82%; Figure 8D). RNAfold analysis then provided an explanation for this unexpected finding: in variant C44/45 8M, formation of the CBCH is favored upon disruption of helix P2 during pRNA synthesis, whereas a hairpin structure forming in the 3’-CB, made possible by the 8M mutations, is favored in the case of the wt 8M RNA (Figure 8A, bottom structures). This finding indicates that an extra hairpin, whenever it forms in the 3’-CB, acts as an impediment to the release from σ^A^-RNAP upon pRNA synthesis. 

**Figure 10:**
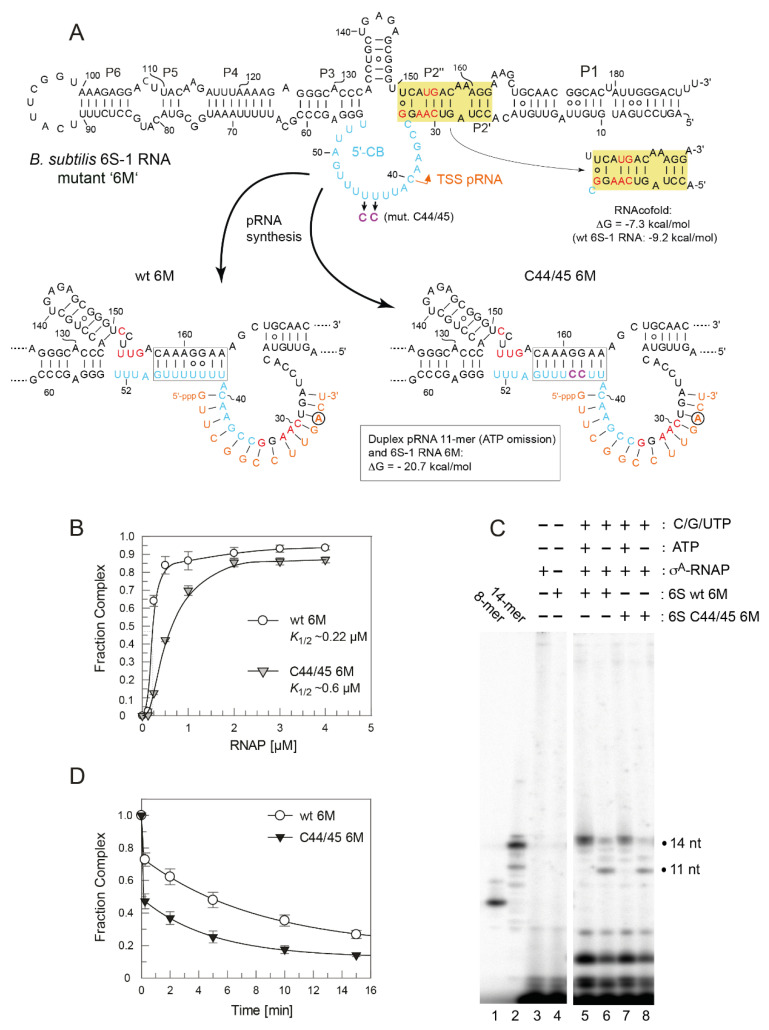
Characterization of 6S-1 RNA variants wt 6M and C44/45 6M (**A**) Secondary structure illustration exactly as in Figure 8A. The six mutations (6M) relative to the wt RNA are highlighted in red. (**B**) σ^A^-RNAP binding affinity for 6S-1 RNA variants wt 6M and C44/45 6M; for more details, see legend to Figure 8B. (**C**) pRNA transcription by SG7 σ^A^-RNAP using the two 6S-1 mutant RNAs as templates, as detailed in the legend to Figure 8C. (**D**) Kinetics of pRNA-induced complex decay of wt 6M or C44/45 6M RNAs using SG7 σ^A^-RNAP (2.6 µM) and 100 µM each CTP, GTP, and UTP. The data were fit to an equation for a double exponential curve with offset. The rate constants *k*_1_ for the fast phase could not be stably predicted, those for the slow phase (*k*_2_) were 0.12 ± 0.02 min^−1^ (wt 6M) and 0.21 ± 0.01 min^−1^ (C44/45 6M), the release-resistant complex fractions were 0.18 (wt 6M) and 0.13 (C44/45 6M).

To avoid formation of this extra hairpin, we constructed variant 6M by reverting C151 and U153 in variant 8M back to U and A residues, respectively (Figure 10A). This slightly reduced the predicted stability of helix P2 from ΔG = −9.2 (wt 6S-1 RNA) to −7.3 kcal/mol. As a consequence, RNAfold dot plot analysis now predicted formation of the CBCH instead of P2 already in the free C44/45 6M mutant RNA, but not in the wt 6M RNA. As before, we compared σ^A^-RNAP affinity of the variants wt 6M and C44/45 6M, again showing an almost three-fold reduction in RNAP affinity owing to the C44/45 mutations (Figure 10B). In the pRNA transcription pattern, 11-mers were prominent under ATP omission conditions, although non-templated 12 to 14-mers appeared as well (Figure 10C, lanes 6 and 8), somewhat more pronounced than in the case of the 8M variants (Figure 8C). Remarkably, the release extent and kinetics were now clearly increased for the 6M mutant in the C44/45 vs. wt background (Figure 10D). Furthermore, the rearranged 6S-1 RNA:pRNA hybrids formed by the 6M variants appeared as more distinct bands on native gels than the 8M mutants (cf. Figure 9A,B), suggesting that a weaker P2 helix formation potential has a stabilizing effect on the 6S-1 RNA:pRNA hybrid structures.

**Figure 11:**
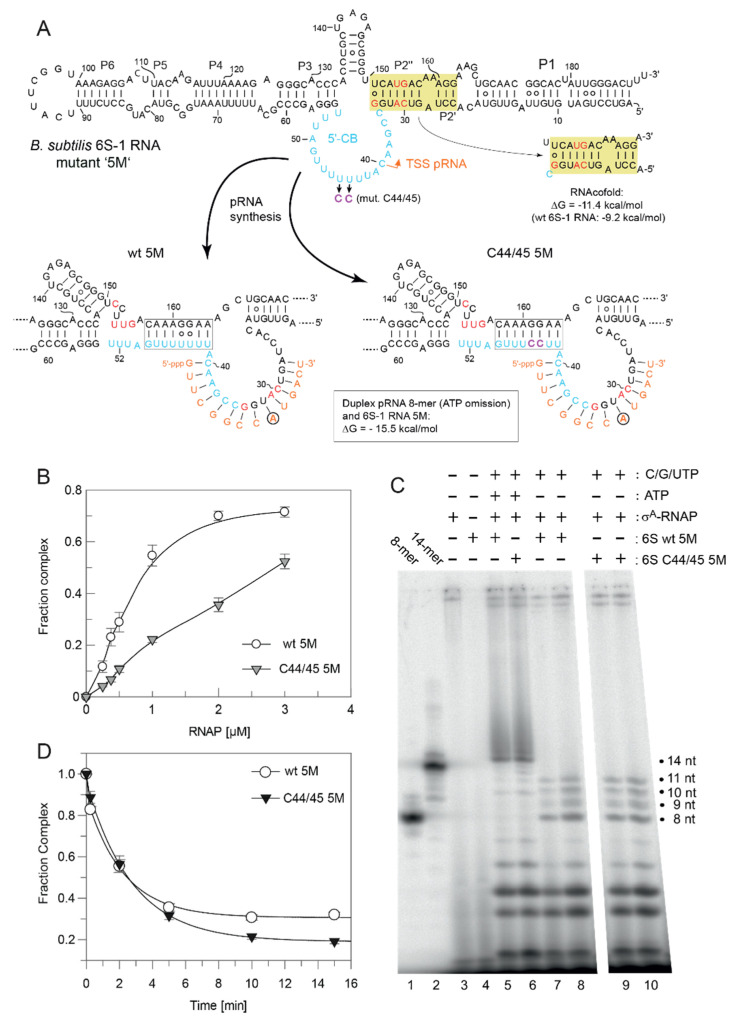
Characterization of 6S-1 RNA variants wt 5M and C44/45 5M (**A**) Secondary structure illustration exactly as in Figure 8A. The five mutations (5M) relative to the wt RNA are highlighted in red. (**B**) σ^A^-RNAP binding affinity for 6S-1 RNA variants wt 5M and C44/45 5M. The lines are spline curves; *K*_1/2_ values were not calculated, as σ^A^-RNAP titrations for variant C44/45 5M did not reach saturation. For more details, see legend to Figure 8B. (**C**) pRNA transcription by SG7 σ^A^-RNAP using the two 6S-1 mutant RNAs as templates, as detailed in the legend to Figure 8C. The difference between lanes 7/8 and 9/10 is the use of 1 µM (lanes 7 and 9) or 2 µM (lanes 8 and 10) enzyme. In lane 7, the total amount of radioactivity was lower than in lanes 8–10, affecting the intensity of all transcription products. (**D**) Kinetics of pRNA-induced decay of 6S-1 RNA:RNAP complexes for wt 5M or C44/45 5M RNAs using SG7 σ^A^-RNAP (2 µM) and 100 µM each CTP, GTP, and UTP. The data were fit to an equation for a double exponential curve with offset. The rate constants *k*_1_ for the fast phase could not be stably predicted, those for the slow phase (*k*_2_) were 0.45 ± 0.06 min^−1^ (wt 5M) and 0.36 ± 0.01 min^−1^ (C44/45 5M), the release-resistant complex fractions were 0.31 (wt 5M) and 0.19 (C44/45 5M).

Finally, we again slightly reinforced helix P2 by reverting A32 to U to restore one internal base pair (variants 5M; Figure 11A; predicted P2 stability: −11.4 vs. −9.2 kcal/mol for the wt RNA). As a consequence, 8-mers were the major pRNA species expected under ATP omission conditions (Figure 11A), although 9 to 11-mers appeared as well (Figure 11C, lanes 7–10; see also Figure S3). These pRNAs formed gel-resolvable complexes with the 5M variants (Figure 9C). For unknown reasons, the 5M design caused a particularly pronounced deterioration of RNAP binding in the C44/45 relative to wt background (Figure 11B). Regarding extent and kinetics of release (complex decay), the wt 5M variant became slightly faster in the slow phase than the C44/45 5M variant (*k*_2_ = 0.45 vs. 0.36 min^−1^), but still formed more release-resistant complexes than the C44/45 5M RNA (0.31 vs. 0.19; Figure 11D).

### 3.3. 6S-1 RNA Refolding and Disruption of the Complex with RNAP Can Be Induced by a Stably Bound Oligonucleotide Hexamer

**Figure 12:**
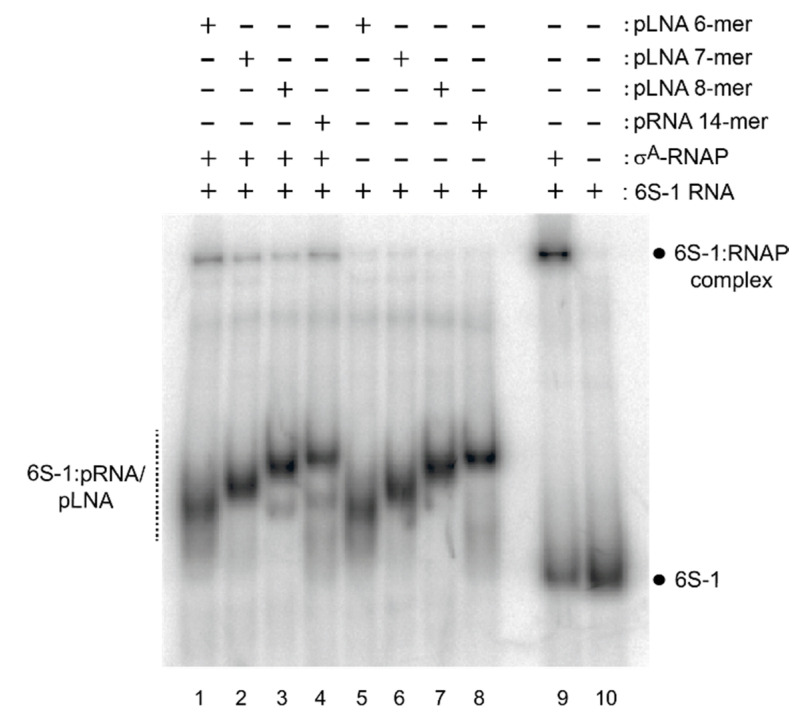
Effect of pLNA/pRNA length on 6S-1 RNA structure analyzed by 10% native PAGE. Annealing of pLNA 6-, 7- or 8-mers, or a pRNA 14-mer, to 5′-^32^P-labeled 6S-1 RNA either without (lanes 5–8) or with native σ^A^-RNAP (lanes 1–4); σ^A^-RNAP was added after pLNA/pRNA annealing to 6S-1 RNA, followed by incubation for 30 min at 37 °C before gel loading. For more details, see Section 2.10.

We previously showed that an iso-sequential analog of a pRNA 8-mer that consists of locked nucleic acid (LNA) residues stably binds to 6S-1 RNA and retards the RNA’s mobility in native PAA gels to a very similar extent as a pRNA 14-mer [12]. Here we addressed the question of whether even iso-sequential pLNA oligonucleotides shorter than 8 nt might be able to induce 6S-1 RNA refolding such that complex formation with σ^A^-RNAP is disrupted/prevented. For this purpose, we annealed chemically synthesized pLNA 6-, 7- and 8-mers to radiolabeled 6S-1 RNA, and analyzed their mobilities by native PAGE. The complex of 6S-1 RNA with the pLNA 8-mer migrated almost as fast as the complex with the pRNA 14-mer used as reference (Figure 12, lines 7 and 8). Gel mobility incrementally decreased for complexes with the pLNA 7-mer and 6-mer (lanes 5 and 6), but the 6S-1:pLNA 6-mer complex still migrated substantially slower than free 6S-1 RNA (Figure 12, cf. lanes 1 and 5 with lanes 9 and 10). As the complex with the pRNA 14-mer, all three 6S-1:pLNA complexes prevented 6S-1 RNA binding to σ^A^-RNAP (cf. lanes 1–4 and lane 9). Our findings demonstrate that already stable binding of a 6-meric pRNA mimic constrains or compacts the 6S-1 RNA conformation to an extent that impedes σ^A^-RNAP binding.

### 3.4. Atomic Force Microscopy Analysis of Free 6S-1 and 6S-1:pLNA Complexes

**Figure 13:**
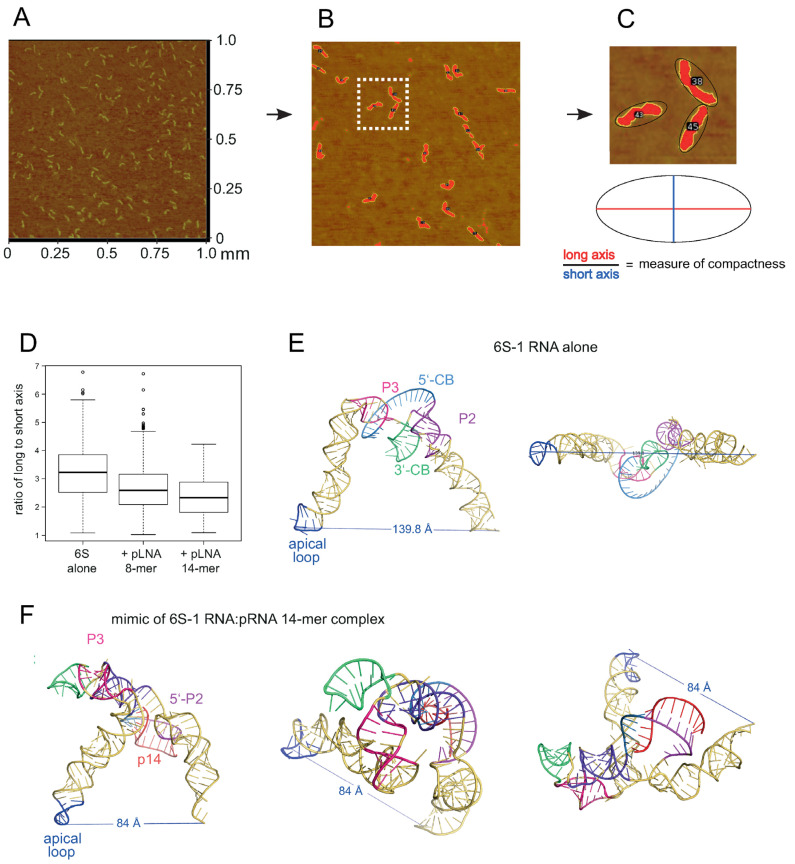
Atomic Force Microscopy (AFM) of *B. subtilis* 6S-1 RNA. (**A**) Raw image (1 × 1 μm area) of 6S-1 RNA molecules spread on a mica surface (for details, see Section 2.11). (**B**) Enlarged image section after image processing with contours of individual molecules encircled by an ellipse. (**C**) Magnified visualization of three encircled example molecules (magnification of the area marked by the white frame in panel B) next to a cartoon of ellipse evaluation. For more individual images considered to be representative based on visual inspection, see Figure S4. (**D**) Boxplots displaying the ratio of long to short axis of ellipses (a value of 1 corresponds to a perfect cycle) for 6S-1 RNA alone or annealed to a pLNA 8- or 14-mer; all residues were locked nucleic acid (LNA) analogs. (**E**,**F**) RNAComposer 3D approximations of 6S-1 RNA in (**E**) its native conformation (as in Figure 1A with unstructured 5’-CB) and (**F**) in its rearranged structure (Figure 1B with extended CBCH) in complex with a pRNA 14-mer. The model for 6S-1 RNA alone is illustrated in two perspectives and that for the 6S-1 RNA:pRNA complex in three perspectives. The secondary structural elements in panels E and F are indicated according to Figure 1; p14, pRNA 14-mer. The distance between the P atom of 6S-1 RNA residue U94 in the apical loop and the 3’-oxygen of the last nucleotide (U190) is depicted as well. For the 2D structure annotations used as templates for RNAcomposer, see Figure S5.

We further employed atomic force microscopy (AFM) to study the shape of free 6S-1 RNA in comparison with complexes annealed to a pLNA 8-mer or 14-mer (Figure 13A,B). Particularly for pRNA 8-mers the use of all-LNA versions was essential to prevent dissociation of annealing complexes within the time frame of the experiment [12]. As a measure for the compactness and possibly for the bend of each 6S RNA molecule, an ellipse was fitted around the detected particle and the ratio of its long and short axis was used for shape estimation (Figure 13C). The AFM particle analysis showed a significant (*p* = 2.2 × 10^−16^, one tailed Welch Two Sample *t*-test) increase in compactness for 6S-1 RNA:pLNA complexes relative to free 6S-1 RNA (Figure 13D). For details on data recording and evaluation, see Materials and Methods.

## 4. Discussion

The *B. subtilis* 6S-1 RNA truncation variant 6S_78_ and 6S_82cp_ with length reductions of almost 60% still showed hallmarks of 6S RNAs, that is, served as template for transcription of pRNAs including 14-mers to form 6S RNA:pRNA hybrid structures that trigger 6S RNA release from σ^A^-RNAP (Figure 2E). The truncations caused a ~10-fold decrease in affinity that may be due to the loss of contacts in the apical stem region where a −35-like contact region to the highly conserved domain 4 of σ^70^/σ^A^-like sigma factors was identified in the *E. coli* system [14]. However, in the *E. coli* system, already truncation of the distal part of the apical stem (deletion of the apical loop plus approx. P6/P5; Figure 1A) completely abolished gel-resolvable binding to σ^A^-RNAP [15]. We conclude that the −35 region is more crucial for *E. coli* 6S RNA interaction with σ^70^-RNAP than for binding of *B. subtilis* 6S-1 RNA to σ^A^-RNAP. Despite the affinity reduction of variants 6S_78_ and 6S_82cp_, our results demonstrate that the CB region and its adjacent short helices include all elements required for basic 6S-1 RNA function. Thus, the native length of the helical arms is not essential for the pRNA-induced disruption of 6S RNA:σ^A^-RNAP complexes.

Previous investigations in the *E. coli* system focused on the ‘−10’-like region of the 3’-CB [14,15,16]. Six tested single mutations at positions 131 to 136 in the 3’-CB of *E. coli* 6S RNA (see Figure S1A) had no or minor effects (~35% affinity reduction) on ground state binding to the σ^70^-RNAP enzyme, of which one (C132A) caused a release defect and another one (U134A) accelerated the release rate [15]. The lack of a binding defect upon mutation of A131, U135, and G136 was confirmed in another study [14]. As nucleotides 132 to 136 form base pairs in the 3’-CB hairpin in *E. coli* 6S RNA:pRNA hybrid structures (see Figure S1A), the just mentioned findings suggest a primary role of these nucleotides in the 6S RNA release mechanism during pRNA synthesis. For *B. subtilis* 6S-1 RNA [12], we observed a two- to three-fold lower affinity for a triple mutant of 6S-1 RNA (C136A/G145U/C146A) that is unable to form the 3’-CB hairpin. We also provided evidence that the triple mutant is conformationally more flexible than the native 6S-1 RNA [12]. So, it seems that the 3’-CB hairpin of *B. subtilis* 6S-1 RNA, predicted by RNAfold to stably form already in the free RNA, is either involved in ground state binding to σ^A^-RNAP or indirectly supports the interaction of other RNA elements with the holoenzyme. We conclude that the 3’-CB interaction with RNAP differs in Enterobacteriaceae such as *E. coli* compared with *B. subtilis* and related Firmicutes. This leads to questions regarding the extent to which the 5’-CB contributes to interactions with RNAP. Unfortunately, residues of the 5’-CB were not resolved in the *E. coli* cryo-EM structure [14] and ground state binding data are not available for *E. coli* 6S RNA variants with mutations in the 5’-CB. Two mutations upstream of the TSS (U44) in the 5’-CB of *E. coli* 6S RNA, namely A52U and A50U, were tested for pRNA-induced release from σ^70^-RNAP [30]. While the A52U variant had no effect on the release rate, the A50U mutant released 2.5-fold slower than the parental 6S RNA. Surprisingly, A50U and A52U in combination were able to rescue the release defect caused by a U44A mutation at the TSS, a finding as yet not understood [30]. For *B. subtilis* 6S-1 RNA, we saw clear binding defects upon mutation of A50 to U, C, or G and U44/45 to C44/45 upstream of the pRNA TSS (C40), consistent with base-specific direct contacts to the enzyme in this part of the 5’-CB. At present, it can also not be excluded that the C44/45 double mutation more indirectly reduced binding affinity through shifting the conformational equilibrium of unbound 6S-1 RNA toward conformers forming the CBCH instead of helix P2. It is noteworthy that two other 6S-1 RNA mutations in the 5’-CB, U47A, and U53A had no effect on ground state binding (Figure S2), indicating that not all base identities in the 5’-CB are crucial for interaction with σ^A^-RNAP.

Stabilization of the CBCH by introducing the C44/45 double mutation that replaced two G:U with G:C pairs (Figure 1) increased the fraction of shorter pRNA in vitro transcripts (11/12-mers; Figure 4B,C), consistent with the notion that the CBCH lowers the activation barrier for pRNA-induced 6S-1 RNA refolding, to an extent depending on its relative stability and probability of formation. In our experimental setup, the kinetics of pRNA-induced 6S-1 RNA release from σ^A^-RNAP (complex decay) remained indistinguishable between the wt and C44/45 mutant RNA upon pRNA synthesis in the presence of all four NTPs (Figure 7). To further carve out differences between the two, we introduced the 8M, 6M, and 5M mutations into helix P2 of the wt and C44/45 6S-1 RNAs. This was conceived to adjust pRNA synthesis under ATP omission conditions to length species of 11 (8M, 6M) or 8 (5M) nucleotides and to compensate this length reduction by increasing the proportion of G:C pairs in 6S-1 RNA:pRNA hybrids. The 8M variants indeed gave rise to the synthesis of pRNA 11-mers under ATP omission conditions (Figure 8C) and to formation of gel-resolvable 6S-1 RNA:pRNA complexes (Figure 9A). The 6S-1 RNA:pRNA hybrid RNA appeared as more distinct bands in the C44/45 relative to the wt background, in line with the stabilizing effect of the CBCH (Figure 9A, cf. right and left gel image). Surprisingly, an enlarged fraction of release-resistant 6S-1 RNA:σ^A^-RNAP complexes was observed in the wt background (Figure 8D). This can be explained by pRNA-induced formation of a second small hairpin in the 3’-CB that is energetically favored in the wt background, but not in the C44/45 context that favors formation of the CBCH (Figure 8A). Formation of the extra hairpin in the 3’-CB may inhibit a conformational change in RNAP that is required for enzyme release, may induce a release-resistant RNAP conformation, or might tighten the 6S-1 RNA:σ^A^-RNAP interaction, thereby locking the complex in a high affinity state. With the 6M variant, we prevented formation of the artificial 3’-CB hairpin and also destabilized helix P2. With the wt and C44/45 6M variants, 6S-1 RNA:pRNA hybrids now appeared as sharp bands with identical mobility in native gels (Figure 9B). The 6M variants gave rise to primarily pRNA 11-mers, but appeared somewhat more permissive than the 8M variant to the synthesis of longer pRNAs despite the omission of ATP (Figure 10C). Here, the release process was faster and more complete in the C44/45 versus wt background (Figure 10D). Again, as for the 8M variants, these findings are in line with the support function of the CBCH in the release process. It cannot be completely ruled out that the 2- to 3-fold decreased ground state binding affinity of the C44/45 6M mutant relative to the wt 6M variant might have contributed to its more efficient release from RNAP. We have attempted to marginalize this possibility by analyzing 6S-1 RNA release in the presence of σ^A^-RNAP concentrations considerably above the *K*_d_ for the C44/45 mutant RNA.

Abortive pRNA transcription results from RNAP scrunching, where the enzyme remains stationary on the template but reels in RNA downstream of the TSS, thereby threads the template RNA strand through the active site for pRNA synthesis. Panchapakesan and Unrau [16] devised a model according to which formation of the extended 3’-CB hairpin upon pRNA synthesis on *E. coli* 6S RNA accumulates additional strain during scrunching, explaining why σ^70^ ejection occurs at a pRNA length of 9 nt on the wt RNA but increases to 14 nt when the hairpin is disrupted. Our observation of a shift to shorter pRNAs with the C44/45 mutant RNA (Figure 4B,C) would be in line with the stabilized CBCH increasing strain during scrunching as well. The 3’-CB hairpin of 6S-1 RNA may also add strain during scrunching, as our 6S-1 triple mutation (C136A/G145U/C146A) that disrupts hairpin formation caused a shift to longer pRNAs including runoff-like transcripts [12]. Increased proportions of runoff-like pRNAs were also observed in vivo for *B. subtilis* 6S-2 RNA [31], which is less structured than 6S-1 RNA in the CB [13]. Thus, scrunching of RNAP holoenzymes and refolding of 6S RNA during pRNA synthesis are likely intertwined components of the release mechanism. A related issue is the pRNA length at which σ^A^/^70^-RNAP switches to the elongation mode. For DNA promoters, promoter escape has been reported to occur at transcript lengths of 9–15 nt [32,33]. For *E. coli* 6S RNA:σ^70^-RNAP complexes, σ^70^ ejection, and by inference entry into the elongation mode, was observed upon synthesis of pRNA 9-mers followed by dissociation of core RNAP:6S RNA complexes at a pRNA length of 13 nt [16]. Assuming a similar length requirement for σ^A^-RNAP:6S-1 RNA complexes to enter the elongation mode associated with σ^A^ ejection, then our 5M variants may be revealing, since pRNA 8-mers were the main products under ATP omission conditions (Figure 11D and Figure S3). Here, the fast phase of complex decay was almost lacking (Figure 11D). Likewise, the fast phase of complex decay was absent in the release kinetics for wt and C44/45 6S-1 RNAs under ATP omission conditions, limiting pRNA transcription to mainly 8-mers as well (Figure 6C). Although still speculative at present, this could mean that the transition to the elongation mode of RNAP has become rate-limiting for fast release in the case of wt, C44/45, wt 5M, and C44/45 5M 6S-1 RNAs under ATP omission conditions that prevent or largely decelerate the synthesis of pRNAs longer than 8-mers.

Another observation of this study is the extension of pRNAs under ATP omission conditions, despite the restriction of templated pRNAs to a length of 11 (variants 8M and 6M) or 8 (5M) nucleotides. These extensions may be due to NTP misincorporation, non-templated addition of NTPs, or utilization of alternative TSSs.

We demonstrated here that already stable binding of a hexameric pLNA mimic substantially retards gel mobility of 6S-1 RNA and alters its structure to an extent that is incompatible with σ^A^-RNAP binding. In native gels, elongated/extended/rod-shaped RNAs migrate faster than branched molecules [34]. A 6-bp helix apparently constricts conformational flexibility in the 5’-CB to an extent that disfavors formation of an elongated structure as adopted by free 6S-1 RNA. We previously showed for *A. aeolicus* 6S RNA that essentially the same gel mobility shift is obtained by annealing a pRNA 15-mer to nucleotides in the 5’-CB/5’-P2 strand compared with an artificial ‘pRNA’ 15-mer annealing to the opposite part of the CB, namely the 3’-strand of P2 and nucleotides in the 3’-CB [22]. This observation indicates that constricting conformational flexibility by duplex formation involving nucleotides of the 3’- or 5’-CB prevents formation of a more elongated 6S RNA structure. Thus, the latter feature seems to be the reason for the observed gel shifts, rather than formation of a specific 6S RNA:pRNA hybrid structure. Increased deviation from an elongated, rod-shaped structure when a pRNA is stably bound was quantitatively ascertained in our AFM particle shape analyses (Figure 13) and also qualitatively evident upon visual inspection of individual molecule shapes (Figure S4). Upon annealing of the pLNA 8- and 14-mers, increasingly bent, thicker, and shorter molecules appeared (often with three protuberances of about equal length) while slim elongated molecules seemed to be more abundant in images of free 6S-1 RNA (Figure S4). This would be in line with the RNAcomposer 3D predictions shown for free 6S-1 RNA and 6S-1 RNA:pRNA 14-mer complexes in Figure 13E,F, the latter predicted to adopt a more compact shape. Of course, the RNAcomposer predictions should be taken as reasonable yet approximate estimates of 6S-1 RNA folds, with a speculative level of structural detail. We are also aware that some subjective interpretations of individual AFM images (Figure S4) are at risk of overstraining the resolution power of AFM images.

Remarkably, *A. aeolicus* 6S RNA with the artificial ‘pRNA’ 15-mer bound to 3’-P2/3’-CB was inefficient in preventing complex formation with σ^A^-RNAP and rather formed aberrantly migrating complexes with the enzyme in native PAA gels [22]. Thus, constriction of conformational flexibility in the CB may be one component of the 6S-1 RNA release mechanism, but other mechanistic components may be required as well. Conceivably, duplex formation with pRNA in the 5’-CB/5’-P2 region may specifically disrupt nearby enzyme contacts to 5’-CB nucleotides upstream of the TSS, possibly including A50 and U44/45 whose mutation was shown here to weaken ground state binding to the enzyme. If so, formation of the CBCH may support disruption of such contacts involving A50 and U44/45, as those residues are part of the CBCH.

In addition to the pleiotropic and complex phenotypes of the C44/45, 8M, 6M, and 5M mutant RNAs, the sensitivity of 6S-1 RNA function to certain mutations (A50B, C44/45, C136A/G145U/C146A [12]) in the CB region supports the view that the 6S-1 RNA wt sequence and structure, particularly in the CB region, provide an optimal balance of all molecular features that are relevant to the different steps of the RNA’s mechanistic cycle, which includes 6S-1 RNA:σ^A^-RNAP ground state binding affinity, coordination of RNAP scrunching, enzyme switch to the elongation mode, pRNA transcript length, conformational constriction/refolding of 6S-1 RNA, and release of 6S-1 RNA:pRNA hybrids from RNAP.

## Figures and Tables

**Table 1:** Strains and plasmids used in this study.

Strain or Plasmid	Genotype	Reference or Source
Strains		
*B. subtilis* His-rpoC, Δ*bsrAB* (SG7)	PY79Δ*bsrA*:spc (Sp^r^), Δ*bsrB*::kan (Km^r^) *rpoC*Ω pYQ52 (Cm^r^)	[24]
*B. subtilis* 110 NA	*trpC2 spo0A3* su^-^	[25]
Plasmids		
pBB1	pUC18::T7-*bsrA*-190-wt, (Amp^r^)	[12]
pGH2	pUC18::T7-*bsrA*-190-U50, (Amp^r^)	This work
pGH3	pUC18::T7-*bsrA*-190-C50, (Amp^r^)	This work
pGH5	pUC18::T7-*bsrA*-78-6S_78_, (Amp^r^)	This work
pGH6	pUC18::T7-*bsrA*-82-6S_82cp_, (Amp^r^)	This work
pGH12	pUC18::T7-*bsrA*-190-UUUUswap (Amp^r^)	This work
pGH15	pUC18::T7-*bsrA*-190-A47, (Amp^r^)	This work
pGH16	pUC18::T7-*bsrA*-190-C44/45, (Amp^r^)	This work
pGH17	pUC18::T7-*bsrA*-190-A53, (Amp^r^)	This work
pAH_P2swap	pUC18::T7-*bsrA*-190-P2swap (Amp^r^)	This work
pSG1	pUC18::T7-*bsrA*-190-G50, (Amp^r^)	This work
pSG2	pUC18::T7-*bsrA*-190-wt 8M, (Amp^r^)	This work
pSG3	pUC18::T7-*bsrA*-190-C44/45 8M, (Amp^r^)	This work
pSG4	pUC18::T7-*bsrA*-190-wt 6M, (Amp^r^)	This work
pSG5	pUC18::T7-*bsrA*-190-C44/45 6M (Amp^r^)	This work
pSG6	pUC18::T7-*bsrA*-190-wt 5M (Amp^r^)	This work
pSG7	pUC18::T7-*bsrA*-190-C44/45 5M (Amp^r^)	This work

## Data Availability

RNAfold http://rna.tbi.univie.ac.at/cgi-bin/RNAWebSuite/RNAfold.cgi (accessed on 23 December 2021). RNAcomposer https://rnacomposer.cs.put.poznan.pl/ (accessed on 15 December 2021).

## Supplementary Materials

The following supporting information can be downloaded at: https://www.mdpi.com/article/10.3390/ncrna8010020/s1, Figure S1: The pRNA-induced structural transition of *E. coli* 6S RNA and sequence identity between *E. coli* 6S RNA and *B. subtilis* 6S-1 RNA; Figure S2: Binding affinity of σ^A^-RNAP to 6S-1 RNA wt and other mutant RNAs; Figure S3: pRNA transcription by SG7 σ^A^-RNAP using 6S RNAs wt 5M and C44/45 5M as templates; Figure S4: Example AFM images of free 6S-1 RNA and 6S-1 RNA complexes with pLNA 8- and 14-mers; Figure S5: Secondary structure dot-bracket notation of free 6S-1 RNA and the 6S-1 RNA:pRNA 14-mer complex used as input for 3D predictions by RNA composer.

## References

[1] Barrick J.E., Sudarsan N., Weinberg Z., Ruzzo W.L., Breaker R.R. (2005). 6S RNA is a widespread regulator of eubacterial RNA polymerase that resembles an open promoter. RNA.

[2] Wassarman K.M., Storz G. (2000). 6S RNA regulates *E. coli* RNA polymerase activity. Cell.

[3] Neusser T., Polen T., Geissen R., Wagner R. (2010). Depletion of the non-coding regulatory 6S RNA in *E. coli* causes a surprising reduction in the expression of the translation machinery. BMC Genom..

[4] Cavanagh A.T., Klocko A.D., Liu X., Wassarman K.M. (2008). Promoter specificity for 6S RNA regulation of transcription is determined by core promoter sequences and competition for region 4.2 of sigma70. Mol. Microbiol..

[5] Cavanagh A.T., Wassarman K.M. (2014). 6S RNA, a global regulator of transcription in *Escherichia coli*, *Bacillus subtilis*, and beyond. Annu. Rev. Microbiol..

[6] Steuten B., Hoch P.G., Damm K., Schneider S., Köhler K., Wagner R., Hartmann R.K. (2014). Regulation of transcription by 6S RNAs: Insights from the *Escherichia coli* and *Bacillus subtilis* model systems. RNA Biol..

[7] Wassarman K.M. (2018). 6S RNA, a Global Regulator of Transcription. Microbiol. Spectr..

[8] Wehner S., Damm K., Hartmann R.K., Marz M. (2014). Dissemination of 6S RNA among bacteria. RNA Biol..

[9] Willkomm D.K., Minnerup J., Hüttenhofer A., Hartmann R.K. (2005). Experimental RNomics in *Aquifex aeolicus*: Identification of small non-coding RNAs and the putative 6S RNA homolog. Nucleic Acids Res..

[10] Nickel A.I., Wäber N.B., Gößringer M., Lechner M., Linne U., Toth U., Rossmanith W., Hartmann R.K. (2017). Minimal and RNA-free RNase P in Aquifex aeolicus. Proc. Natl. Acad. Sci. USA.

[11] Burenina O.Y., Elkina D.A., Migur A.Y., Oretskaya T.S., Evguenieva-Hackenberg E., Hartmann R.K., Kubareva E.A. (2020). Similarities and differences between 6S RNAs from *Bradyrhizobium japonicum* and *Sinorhizobium meliloti*. J. Microbiol..

[12] Beckmann B.M., Hoch P.G., Marz M., Willkomm D.K., Salas M., Hartmann R.K. (2012). A pRNA-induced structural rearrangement triggers 6S-1 RNA release from RNA polymerase in *Bacillus subtilis*. EMBO J..

[13] Burenina O.Y., Hoch P.G., Damm K., Salas M., Zatsepin T.S., Lechner M., Oretskaya T.S., Kubareva E.A., Hartmann R.K. (2014). Mechanistic comparison of *Bacillus subtilis* 6S-1 and 6S-2 RNAs—Commonalities and differences. RNA.

[14] Chen J., Wassarman K.M., Feng S., Leon K., Feklistov A., Winkelman J.T., Li Z., Walz T., Campbell E.A., Darst S.A. (2017). 6S RNA Mimics B-Form DNA to Regulate *Escherichia coli* RNA Polymerase. Mol. Cell.

[15] Shephard L., Dobson N., Unrau P.J. (2010). Binding and release of the 6S transcriptional control RNA. RNA.

[16] Panchapakesan S.S., Unrau P.J. (2012). *E. coli* 6S RNA release from RNA polymerase requires sigma70 ejection by scrunching and is orchestrated by a conserved RNA hairpin. RNA.

[17] Wassarman K.M., Saecker R.M. (2006). Synthesis-mediated release of a small RNA inhibitor of RNA polymerase. Science.

[18] Beckmann B.M., Burenina O.Y., Hoch P.G., Kubareva E.A., Sharma C.M., Hartmann R.K. (2011). In vivo and in vitro analysis of 6S RNA-templated short transcripts in *Bacillus subtilis*. RNA Biol..

[19] Murray H.D., Schneider D.A., Gourse R.L. (2003). Control of rRNA expression by small molecules is dynamic and nonredundant. Mol. Cell.

[20] Cabrera-Ostertag I.J., Cavanagh A.T., Wassarman K.M. (2013). Initiating nucleotide identity determines efficiency of RNA synthesis from 6S RNA templates in *Bacillus subtilis* but not *Escherichia coli*. Nucleic Acids Res..

[21] Wurm R., Neußer T., Wagner R. (2010). 6S RNA-dependent inhibition of RNA polymerase is released by RNA-dependent synthesis of small de novo products. Biol. Chem..

[22] Köhler K., Duchardt-Ferner E., Lechner M., Damm K., Hoch P.G., Salas M., Hartmann R.K. (2015). Structural and mechanistic characterization of 6S RNA from the hyperthermophilic bacterium *Aquifex aeolicus*. Biochimie.

[23] Sogo J.M., Inciarte M.R., Corral J., Viñuela E., Salas M. (1979). RNA polymerase binding sites and transcription map of the DNA of *Bacillus subtilis* phage phi29. J. Mol. Biol..

[24] Ganapathy S., Wiegard J.C., Hartmann R.K. (2021). Rapid preparation of 6S RNA-free *B. subtilis* σ^A^-RNA polymerase and σ^A^. J. Microbiol. Methods.

[25] Muñoz-Espín D., Daniel R., Kawai Y., Carballido-López R., Castilla-Llorente V., Errington J., Meijer W.J., Salas M. (2009). The actin-like MreB cytoskeleton organizes viral DNA replication in bacteria. Proc. Natl. Acad. Sci. USA.

[26] Bussiek M., Schöne A., Nellen W., Hartmann R.K., Bindereif A., Schön A., Westhof E. (2014). Atomic Force Microscopy Imaging and Force Spectroscopy of RNA. Handbook of RNA Biochemistry.

[27] Schneider C.A., Rasband W.S., Eliceiri K.W. (2012). NIH Image to ImageJ: 25 years of image analysis. Nat. Methods.

[28] Lorenz R., Bernhart S.H., Hoener zu Siederdissen C., Tafer H., Flamm C., Stadler P.F., Hofacker I.L. (2011). ViennaRNA Package 2.0. Algorithms Mol. Biol..

[29] Popenda M., Szachniuk M., Antczak M., Purzycka K.J., Lukasiak P., Bartol N., Blazewicz J., Adamiak R.W. (2012). Automated 3D structure composition for large RNAs. Nucleic Acids Res..

[30] Oviedo Ovando M., Shephard L., Unrau P.J. (2014). In vitro characterization of 6S RNA release-defective mutants uncovers features of pRNA-dependent release from RNA polymerase in *E. coli*. RNA.

[31] Hoch P.G., Schlereth J., Lechner M., Hartmann R.K. (2016). *Bacillus subtilis* 6S-2 RNA serves as a template for short transcripts in vivo. RNA.

[32] Revyakin A., Liu C., Ebright R.H., Strick T.R. (2006). Abortive initiation and productive initiation by RNA polymerase involve DNA scrunching. Science.

[33] Lee J., Borukhov S. (2016). Bacterial RNA Polymerase-DNA Interaction-The Driving Force of Gene Expression and the Target for Drug Action. Front. Mol. Biosci..

[34] Riesner D., Steger G., Hartmann R.K., Bindereif A., Schön A., Westhof E. (2014). Temperature-gradient gel electrophoresis of RNA. Handbook of RNA Biochemistry.

