# Supplementary material for: Structural and Functional Insight into the Mechanism of Bacillus subtilis 6S-1 RNA Release from RNA Polymerase
Source: Noncoding RNA. 2022 Feb 16;8(1):20. doi: 10.3390/ncrna8010020 (PMC8876501; doi:10.3390/ncrna8010020)
Supplement: Supplementary file 1 [file ncrna-08-00020-s001.zip › Ganapathy_Supplement R2/Fig_S1_030222.pdf]

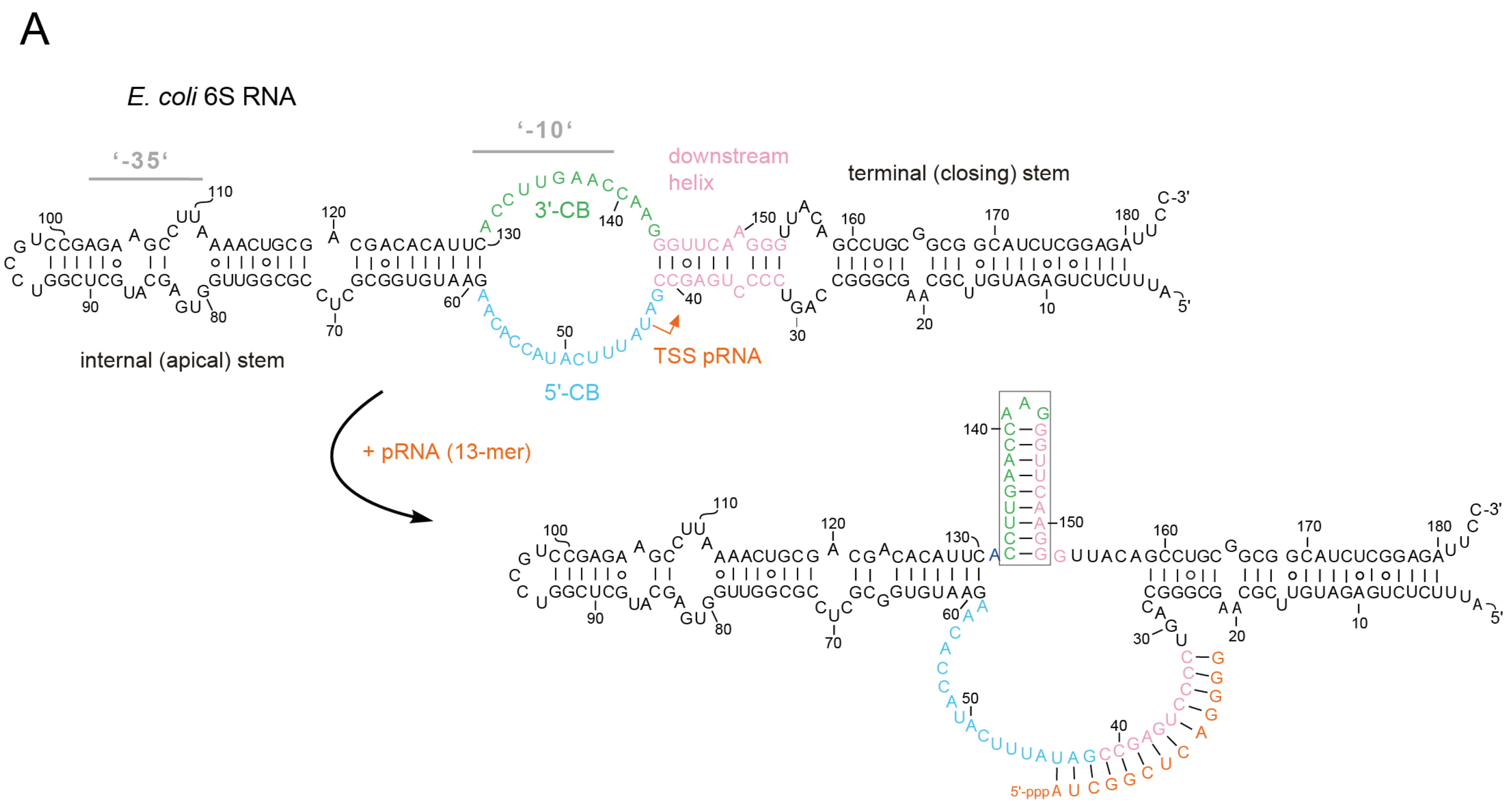

Figure S1: (A) The pRNA-induced structural transition of *E. coli* 6S RNA and (B) sequence identity between *E. coli* 6S RNA and *B. subtilis* 6S-1 RNA. (**A**) Adapted from [12,14]. For more details on abbreviations and color code, see Figure 1 of the main text. (**B**) *Top*: sequence identity between *E. coli* 6S RNA and *B. subtilis* 6S-1 RNA based on the 6S RNA seed alignment in the Rfam database (Rfam 14.7, RF00013; I. Kalvari et al., 2017, *Nucleic Acids Res.*, doi: 10.1093/nar/gkx1038); *bottom*: sequence identity predicted by ClustalW pairwise alignment (J.D. Thompson et al., 2002, *Curr. Protoc. Bioinformatics*; Chapter 2:Unit 2.3; doi: 10.1002/0471250953.bi0203s00).

B

Sequence identity between between *E. coli* 6S RNA and *B. subtilis* 6S-1 RNA based on the Rfam 6S RNA seed alignment

```
Eco  AUUUCUCUGAGAUGUUCGCAAGCGGGCCAGUCCCCUGAGCCGAUAUUUCAUACCACAAGAAUGUGGCGCUCCGCGGUUGGUGAGCAUGCUCGG-----UCC
Bsu  AAAGUCCUGAUGUGUUAGUUGUACACCUA-GUUU-UGA-CCGAACAUUUUUUUGAUUUGGGAGCCCGCAUUUUUAAAUGGCGUACAUGCCUCUUUUCAUUC

Eco  -GU--CCGAGAAGCCUUAAAACUGCGACGACACAUUCACCUUG-AACCAAGGGUUCAAGGGU---UACAGCCUGCGGCGGCAU-CUCGGAGAUUCC 184 nt
Bsu  GGUAAAGAGGACUUACAAGAUUUAAAAGAGGGCACCCACCUGCUGAGAGCGGGUUCA-AAACAAAGGAAAGCUGCAACGGCACUAUUGGGACUUUA 193 nt
```

Eco: E. coli 6S RNA (GenBank accession no. X01238.1); *Bsu*: *B. subtilis* 6S-1 RNA (GenBank accession no. AL009126.3, nt 2814682-2814490)

Identical nucleotides are marked by red letters / Alignment length: 197 nt / Sequence identity: 74 nt --> 74/197 = 37.6% --> average identity: ~ 38%

ClustalW (2.1) pairwise alignment of *E. coli* 6S RNA and *B. subtilis* 6S-1 RNA sequences

```
Eco    AUUUCUCUGAGAUGUUCGCAAGCGGGCCAGUCCCCUGAGCCGAUAUUUCAUACCACAAGA
Bsu    AAAGUCCUGAUGUGUUAGUUGUACACCUAGUU--UUGACCGAACAUUUUUUUGAUUUGGG
       *     ****  **** *        * ***    *** *  * ****  *       *


Eco    AUGUGGCGCUCCGCGGUUGGUGAGCAUGC-UCGGUCCGUCCG----AGAAGCCUUAAAA-
Bsu    AGCCCGCAUUUU-UAAAUGGCGUACAUGCCUCUUUUCAUUCGGUAAAGAGGACUUACAAG
       *    **  *       *** *  ***** **  * * * **    *** * **** **


Eco    --CUGCGACGACACAUUCACCU-UGAACCAAGGGUUCAAGGGUUAC--AGCCUGCGGCGG
Bsu    AUUUAAAAGAGGGCACCCACCUGCUGAGAGCGGGUUCAAAACAAAGGAAAGCUGCAACGG
          *   *     **  *****    *    ********     *   *  ****  ***


Eco    CAUCUCGGAGAUUCC-  184 nt
Bsu    CACUAUUGGGACUUUA  193 nt
       **     * ** *
```

Alignment length: 196 nt / Sequence identity: 88 nt --> 88/196 = 44.9% --> average identity: ~ 45%
