# Supplementary material for: Structural and Functional Insight into the Mechanism of Bacillus subtilis 6S-1 RNA Release from RNA Polymerase
Source: Noncoding RNA. 2022 Feb 16;8(1):20. doi: 10.3390/ncrna8010020 (PMC8876501; doi:10.3390/ncrna8010020)
Supplement: Supplementary file 1 [file ncrna-08-00020-s001.zip › Ganapathy_Supplement R2/Fig_S2_210121.pdf]

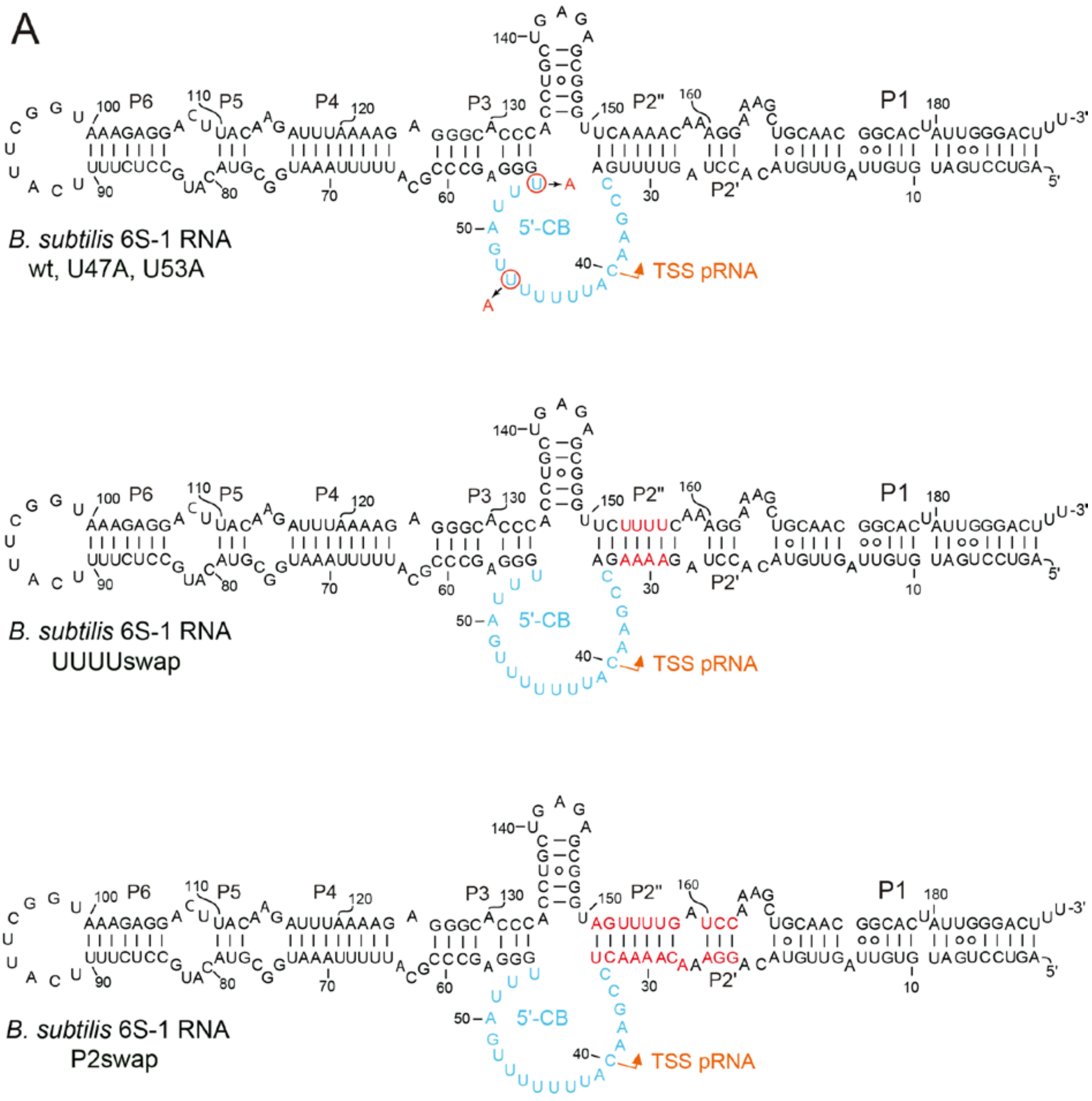
A
P6
P5
P4
P3
P2"
P2'
P1
5'-CB
TSS pRNA
5'
3'
B. subtilis 6S-1 RNA
wt, U47A, U53A
B. subtilis 6S-1 RNA
UUUUswap
B. subtilis 6S-1 RNA
P2swap

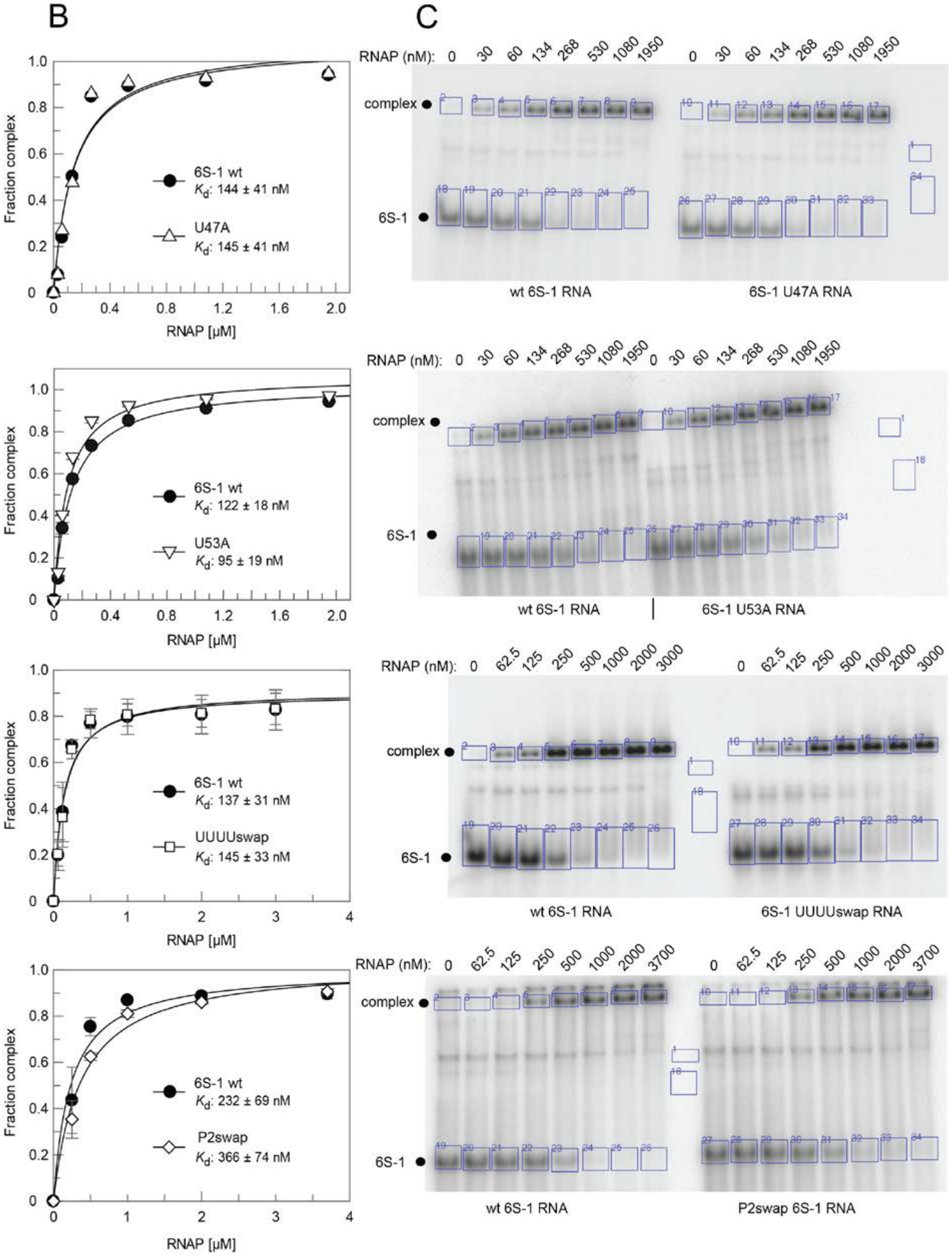

B
C
Fraction complex
RNAP [µM]
6S-1 wt
K_d: 144 ± 41 nM
U47A
K_d: 145 ± 41 nM
RNAP (nM): 0 30 60 134 268 530 1080 1950 0 30 60 134 268 530 1080 1950
complex
6S-1
wt 6S-1 RNA
6S-1 U47A RNA
6S-1 wt
K_d: 122 ± 18 nM
U53A
K_d: 95 ± 19 nM
RNAP (nM): 0 30 60 134 268 530 1080 1950 0 30 60 134 268 530 1080 1950
complex
6S-1
wt 6S-1 RNA
6S-1 U53A RNA
6S-1 wt
K_d: 137 ± 31 nM
UUUUswap
K_d: 145 ± 33 nM
RNAP (nM): 0 62.5 125 250 500 1000 2000 3000 0 62.5 125 250 500 1000 2000 3000
complex
6S-1
wt 6S-1 RNA
6S-1 UUUUswap RNA
6S-1 wt
K_d: 232 ± 69 nM
P2swap
K_d: 366 ± 74 nM
RNAP (nM): 0 62.5 125 250 500 1000 2000 3700 0 62.5 125 250 500 1000 2000 3700
complex
6S-1
wt 6S-1 RNA
P2swap 6S-1 RNA

Figure S2: Binding affinity of σ^A-RNAP to 6S-1 RNA wt and other mutant RNAs. (**A**) Secondary structure presentation of wt 6S-1 RNA and mutants U47A and U53A thereof (top), mutant RNA UUUUswap (middle) and RNA P2swap (bottom), with mutations highlighted in red letters. (**B**) 6S-1 RNA:σ^A-RNAP binding affinity plotted as the fraction of complex as a function of σ^A-RNAP concentration. The data was fit to an equation for a single ligand binding site using Grafit version 5.0.13; data point errors are standard errors of the mean (SEM) and calculated K_d values are depicted within the graphs. Data for the U47A and U53A mutants are based on two and those for mutants UUUUswap and P2 swap on three individual experiments. (**C**) Phosphorimages of exemplary gels with quantification boxes used for K_d determination; for the example at the top, box 1 pixels were subtracted as background from boxes 2 to 17, and box 34 was subtracted from boxes 18 to 33; for the wt 6S-1 RNA panel, the calculated value for (box 2 - box 1)/[(box 2 - box 1) + (box 18 - box 34)] was defined as 0 complex formation and subtracted from the "Fraction complex" values for the corresponding lanes with boxes 3/19, 4/20, 5/21, 6/22, 7/23, 8/24 and 9/25. For more details, see Materials and Methods of the main text.
