# Supplementary material for: Structural and Functional Insight into the Mechanism of Bacillus subtilis 6S-1 RNA Release from RNA Polymerase
Source: Noncoding RNA. 2022 Feb 16;8(1):20. doi: 10.3390/ncrna8010020 (PMC8876501; doi:10.3390/ncrna8010020)
Supplement: Supplementary file 1 [file ncrna-08-00020-s001.zip › Ganapathy_Supplement R2/Fig_S3_210122.pdf]

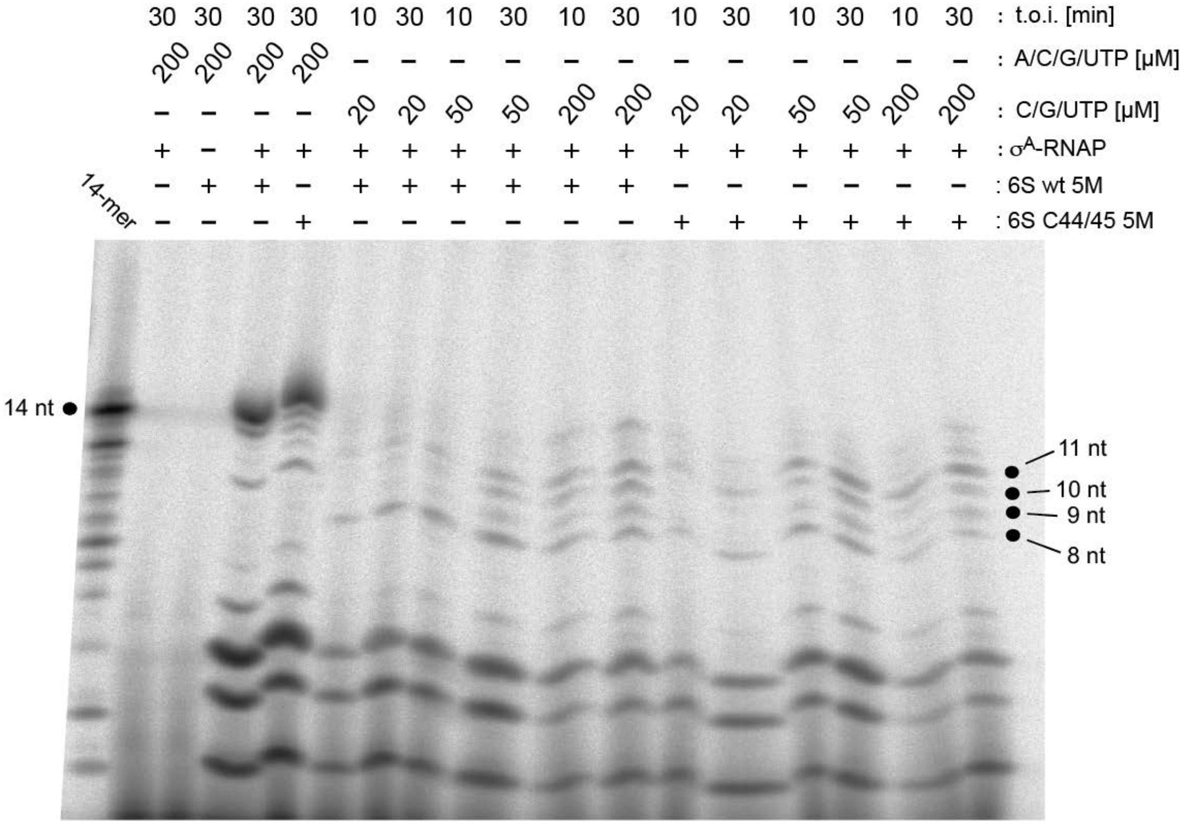

Figure S3: pRNA transcription by SG7 σ^{A}-RNAP using 6S RNAs wt 5M and C44/45 5M as templates. The figure illustrates that the pRNA 8-mer appears first after short incubation (10 min) and at low C/G/UTP concentrations (20 µM each), but longer pRNA products appear under ATP omission conditions upon longer incubation periods and at higher nucleotide concentrations. The longer products can be explained by misincorporation C, G or U instead of the encoded A at position 9. For more details see Fig. 11 and Materials and Methods of the main text.
