# Supplementary material for: Structural and Functional Insight into the Mechanism of Bacillus subtilis 6S-1 RNA Release from RNA Polymerase
Source: Noncoding RNA. 2022 Feb 16;8(1):20. doi: 10.3390/ncrna8010020 (PMC8876501; doi:10.3390/ncrna8010020)
Supplement: Supplementary file 1 [file ncrna-08-00020-s001.zip › Ganapathy_Supplement R2/Fig_S4_AFM_210122.pdf]

6S-1 RNA alone (image #186131): slim elongated molecules #1 and 2 were primarily observed with free 6S-1 RNA

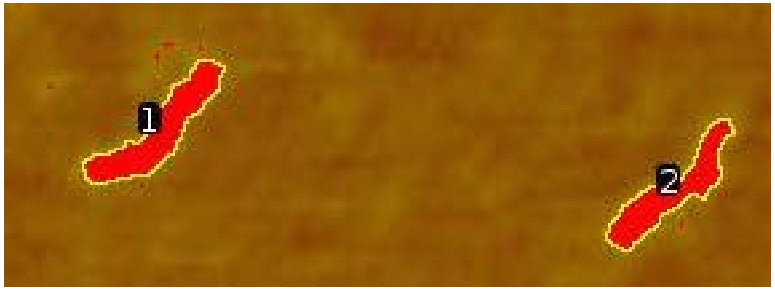

6S-1 RNA alone (image #2711123), strongly bent molecules (#133) rarely observed:

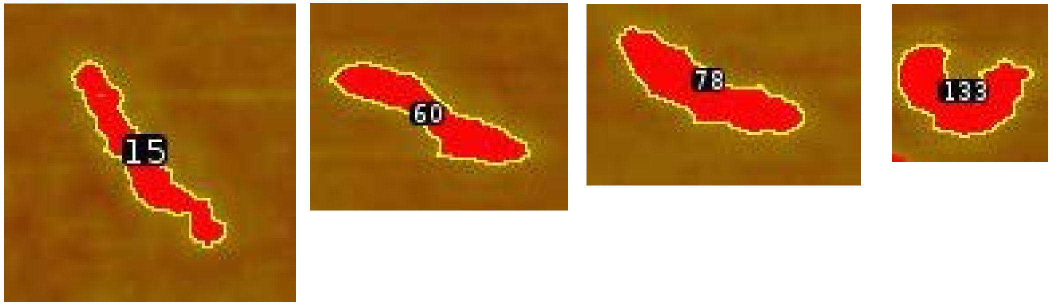

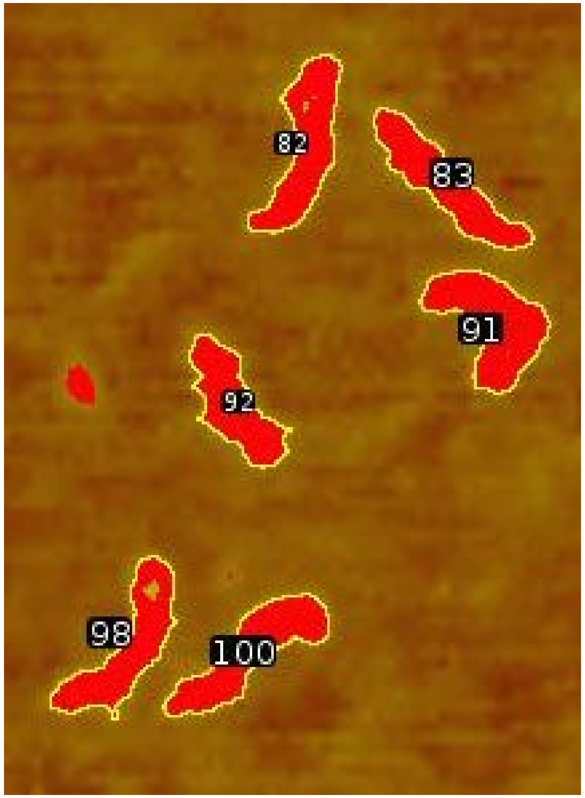

6S-1 RNA alone (image #2711124), other examples:

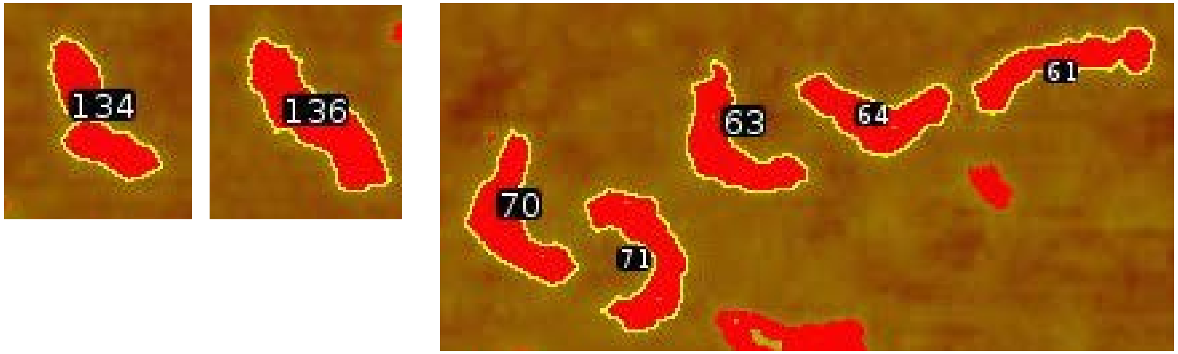

6S-1 RNA alone (image #2711126), other examples; shape of molecules #56 and 106 rarely observed:

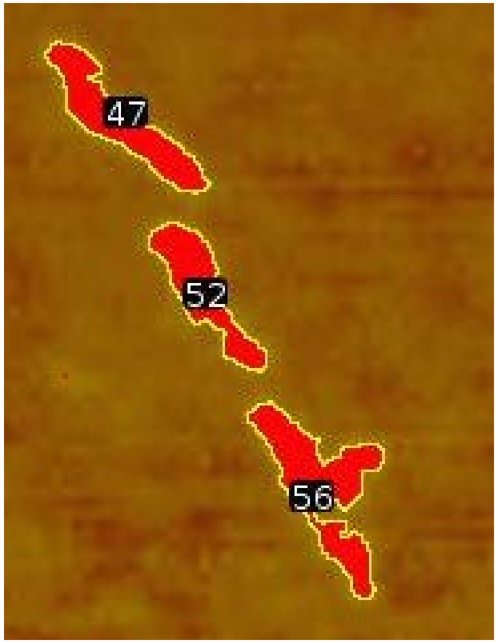

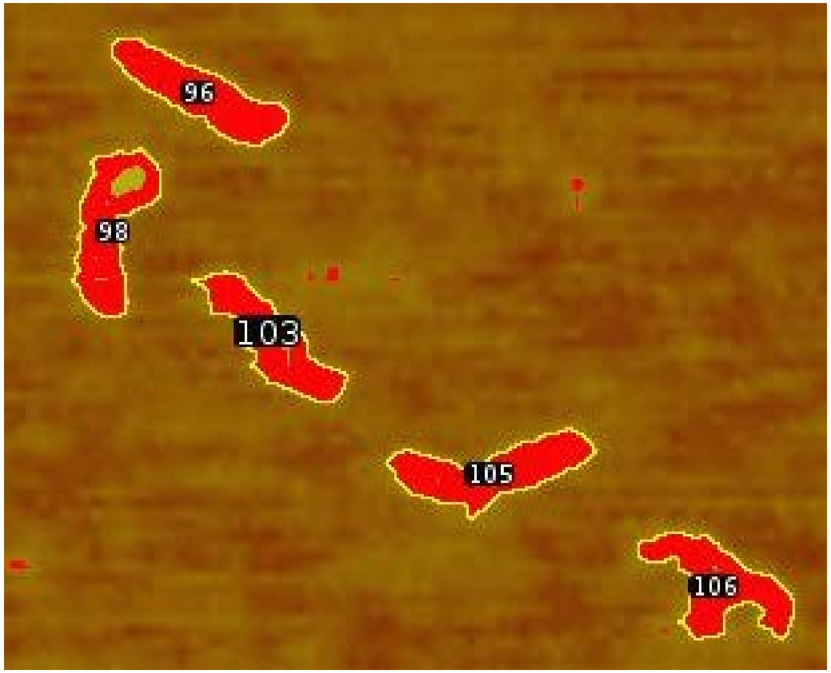

6S-1 RNA complexes with pLNA 14-mer (image #186133) appear mostly thicker when elongated (molecules #1 und 8), only few slim molecules (#15):

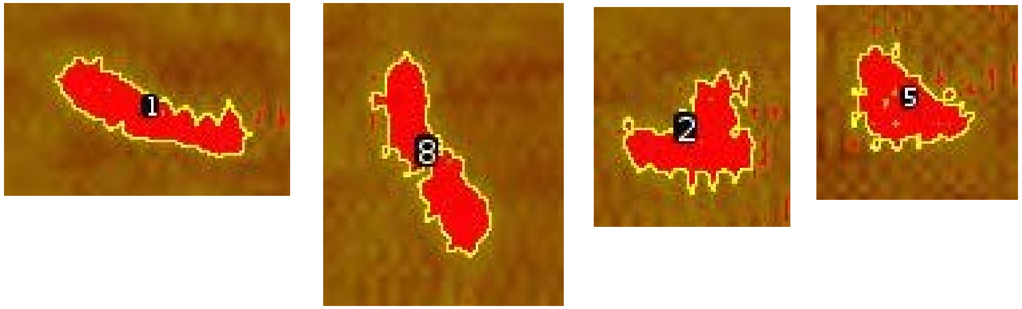

6S-1 RNA complexes with pLNA 14-mer (Image #186139) reveals thicker molecules with three protuberances (#9, 15, 16, 23, 24)

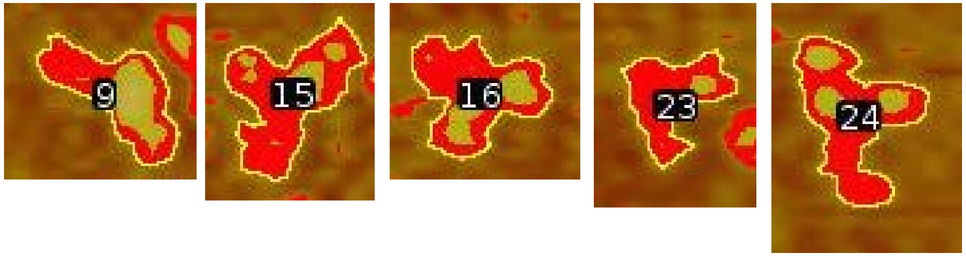

or molecules #9 and 13 in image #186137:

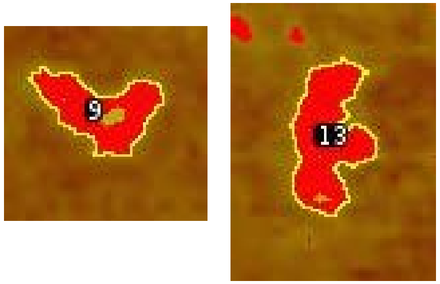

or molecules #8, 9, 10 (image #186134), molecule #4 strongly bent:

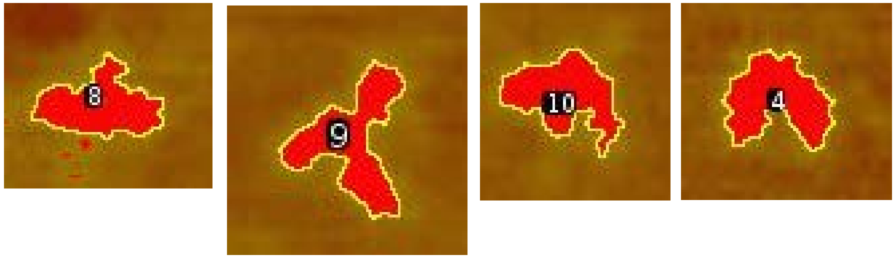

6S-1 RNA complexes with pLNA 8-mer (#27111211)

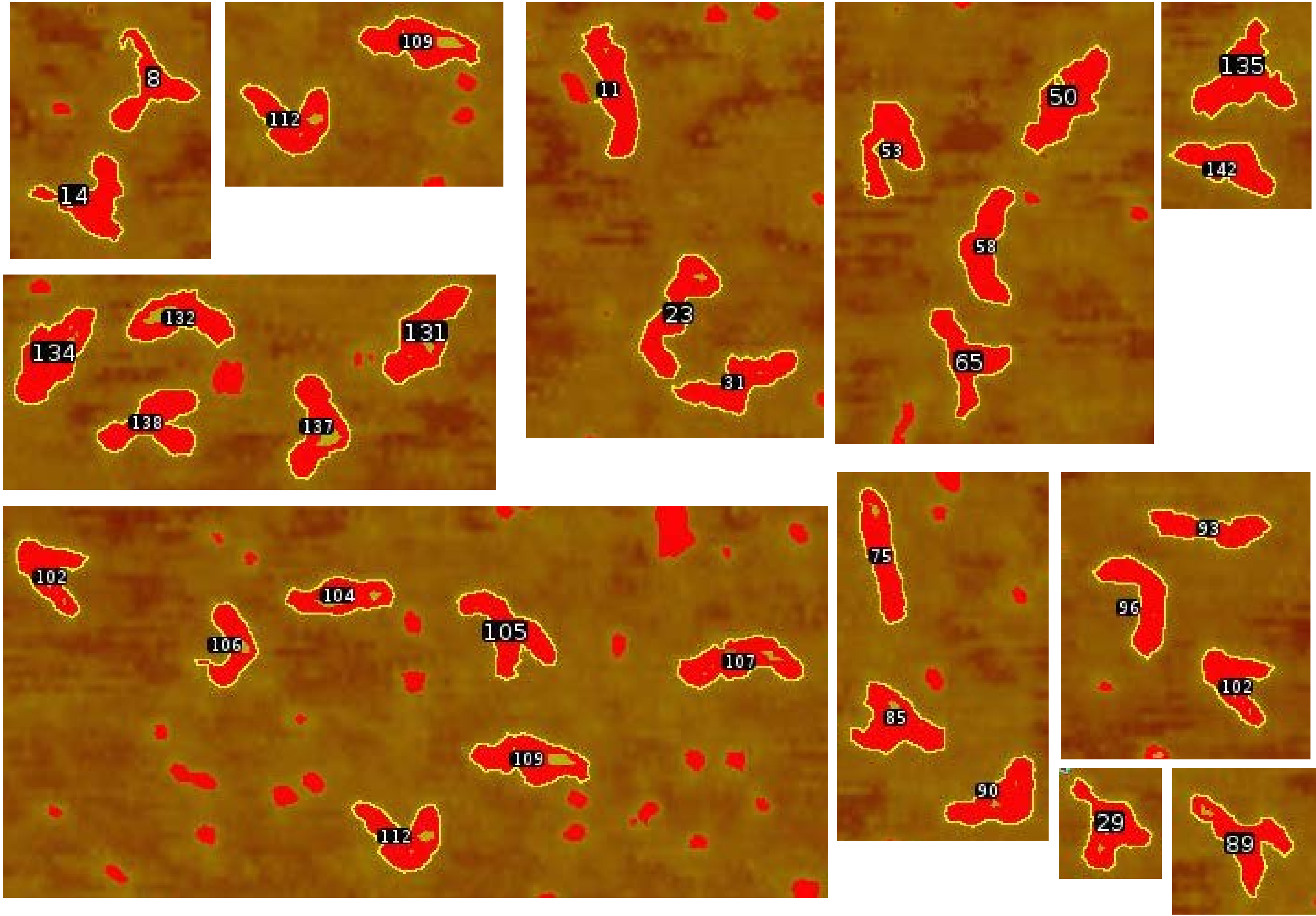

6S-1 RNA complexes with pLNA 8-mer (#27111210)

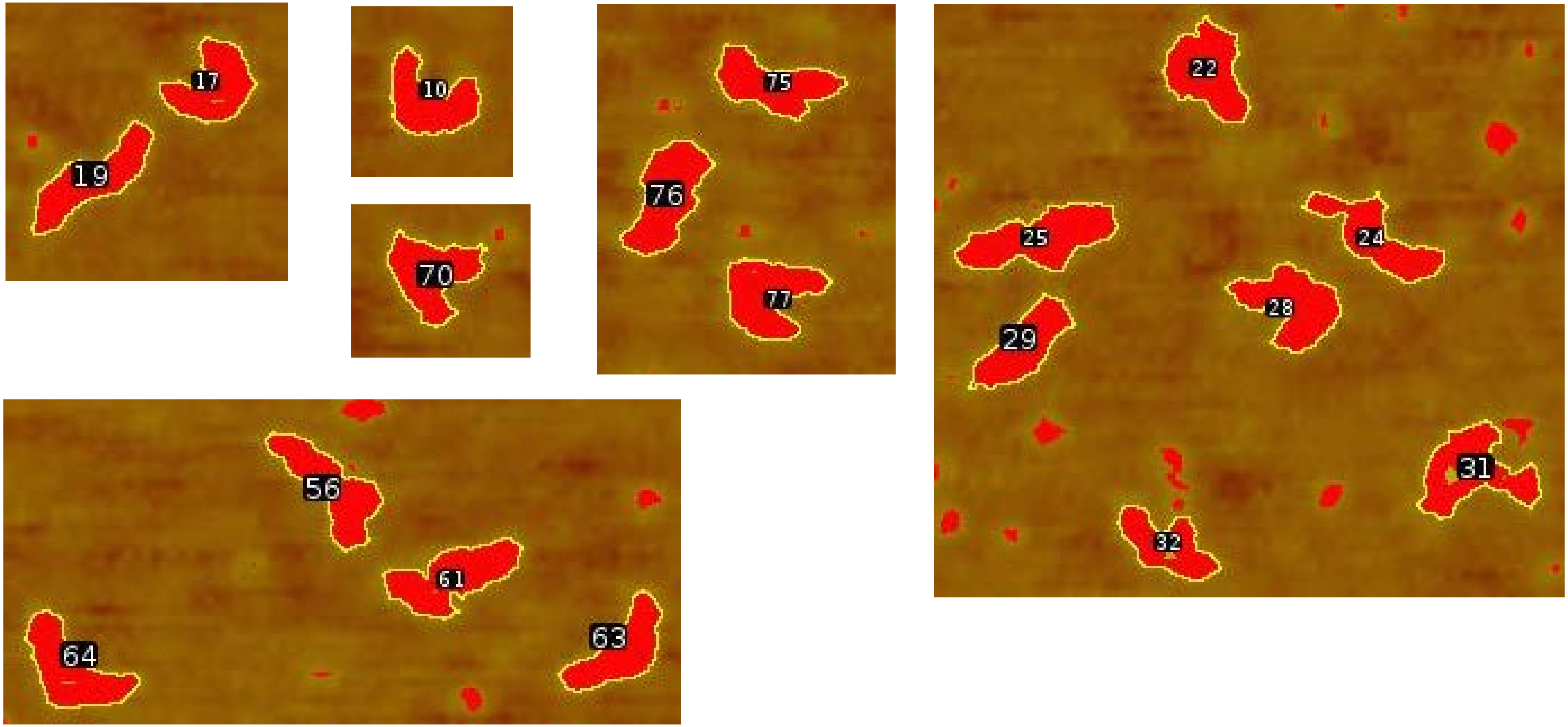

Figure S4. Example AFM images of free 6S-1 RNA and 6S-1 RNA complexes with pLNA 8- and 14-mers. The overall impression (upon visual inspection) is that upon annealing of the pLNA 8- and 14-mers, increasingly bent, thicker and shorter molecules appear, often with three protuberances of about equal lengths. Slim elongated molecules are more abundant in images of free 6S-1 RNA. These trends are in line with the results of the automated, quantitative molecule shape analysis presented in Fig. 13D.
