# Supplementary material for: Structural and Functional Insight into the Mechanism of Bacillus subtilis 6S-1 RNA Release from RNA Polymerase
Source: Noncoding RNA. 2022 Feb 16;8(1):20. doi: 10.3390/ncrna8010020 (PMC8876501; doi:10.3390/ncrna8010020)
Supplement: Supplementary file 1 [file ncrna-08-00020-s001.zip › Ganapathy_Supplement R2/Fig_S5_210122.pdf]

A

Free 6S-1 RNA

6S-1 RNAfold_Centroid_structure (37°C, default parameters), A134-U150 unpaired, 5’-CB unstructured

AGUCCUGAUGUGUUAGUUGUACACCUAGUUUUGACCGAACAUUUUUUUGAUUUGGGAGCCCGCAUUUUUAAAUGGCGUACAUGCCUCUUUUCAUUCGGUAAAGAGGACUUACAAGAUUUAAAAGAGGGCACC

CACCUGCUGAGAGCGGGUUCAAAACAAAGGAAAGCUGCAACGGCACUAUUGGGACUUU
((((((((((((..((((((..(((.(((((((..................(((.((((...(((((((((...(((....((((((((((....)).))))))))...)))...))))))))).)))).))
).((((((...)))))).)))))))..))).....)))))).)))).))))))))))..

Color code: **P2**, **5'-CB**, **P3**,**3'-CB_HP**,

B

6S-1 RNA:pRNA 14-mer complex

6S-1 RNA pRNA 14-mer pseudoknot, Linker 25 x C, CBCH long, A134-U150 unpaired

GUUCGGUCAAAACUCCCCCCCCCCCCCCCCCCCCCCCCCAGUCCUGAUGUGUUAGUUGUACACCU**AGUUUUGACCGAAC**A**UUUUUUUGAUUUGGGAGCCCG**CAUUUUUAAAUGGCGUACAUGCCUCUUUUCA
UUCGGUAAAGAGGACUUACAAGAUUUAAAAGA**GGGCACCC**A**CCUGCUGAGAGCGGG**UUC**AAAACAAAGGAA**AGCUGCAACGGCACUAUUGGGACUUU
[[[[[[[[[[[[[[.........................((((((((((((((.((((((.....]]]]]]]]]]]]]]].((((((((.((((((.((((...(((((((((...(((....(((((((((.
...)).))))))))...)))...))))))))).)))).))).((((((...))))))...))).))))))))...))))))))))))).))))))))))..

Color code: **pRNA 14-mer**, **pRNA target sequence**, **P3**, **3'-CB_HP**, **CBCH_long**

artificial linker sequence (25 C residues); [[...]], bracket annotation for artificial pseudoknot

Figure S4: Secondary structure dot-bracket notation of (A) free 6S-1 RNA and (B) the 6S-1 RNA:pRNA 14-mer complex that were used as templates for 3D predictions by RNAcomposer. For colored structural elements, see Fig. 1 of the main text. For RNAcomposer prediction of the 6S-1:pRNA complex, the 6S-1 RNA sequence was extended at the 5'-end by a linker sequence (here 25 C residues) and the pRNA sequence; very similar results were obtained with U25, A25 or G25 linkers and longer homopolymeric linkers of 50, 75 or 100 nt.
